# A new method to design energy-conserving surrogate models for the coupled, nonlinear responses of intervertebral discs

**DOI:** 10.1007/s10237-023-01804-4

**Published:** 2024-01-20

**Authors:** Maria Hammer, Tizian Wenzel, Gabriele Santin, Laura Meszaros-Beller, Judith Paige Little, Bernard Haasdonk, Syn Schmitt

**Affiliations:** 1 Institute for Modelling and Simulation of Biomechanical Systems, University of Stuttgart, Stuttgart, Germany; 2 Stuttgart Center for Simulation Science (SC SimTech), University of Stuttgart, Stuttgart, Germany; 3 Institute for Applied Analysis and Numerical Simulation, University of Stuttgart, Stuttgart, Germany; 4 Digital Society Center, Fondazione Bruno Kessler, Trento, Italy; 5 Biomechanics and Spine Research Group, School of Mechanical, Medical and Process Engineering, Queensland University of Technology, Brisbane, Australia

**Keywords:** Biomechanics, Intervertebral disc, Kernel approximation, Spine modeling, Elastic surrogates

## Abstract

The aim of this study was to design physics-preserving and precise surrogate models of the nonlinear elastic behaviour of an intervertebral disc (IVD). Based on artificial force-displacement data sets from detailed finite element (FE) disc models, we used greedy kernel and polynomial approximations of second, third and fourth order to train surrogate models for the scalar force-torque -potential. Doing so, the resulting models of the elastic IVD responses ensured the conservation of mechanical energy through their structure. At the same time, they were capable of predicting disc forces in a physiological range of motion and for the coupling of all six degrees of freedom of an intervertebral joint. The performance of all surrogate models for a subject-specific L4$\vert$5 disc geometry was evaluated both on training and test data obtained from uncoupled (one-dimensional), weakly coupled (two-dimensional), and random movement trajectories in the entire six-dimensional (6d) physiological displacement range, as well as on synthetic kinematic data. We observed highest precisions for the kernel surrogate followed by the fourth-order polynomial model. Both clearly outperformed the second-order polynomial model which is equivalent to the commonly used stiffness matrix in neuro-musculoskeletal simulations. Hence, the proposed model architectures have the potential to improve the accuracy and, therewith, validity of load predictions in neuro-musculoskeletal spine models.

## Introduction

**Fig. 1:**
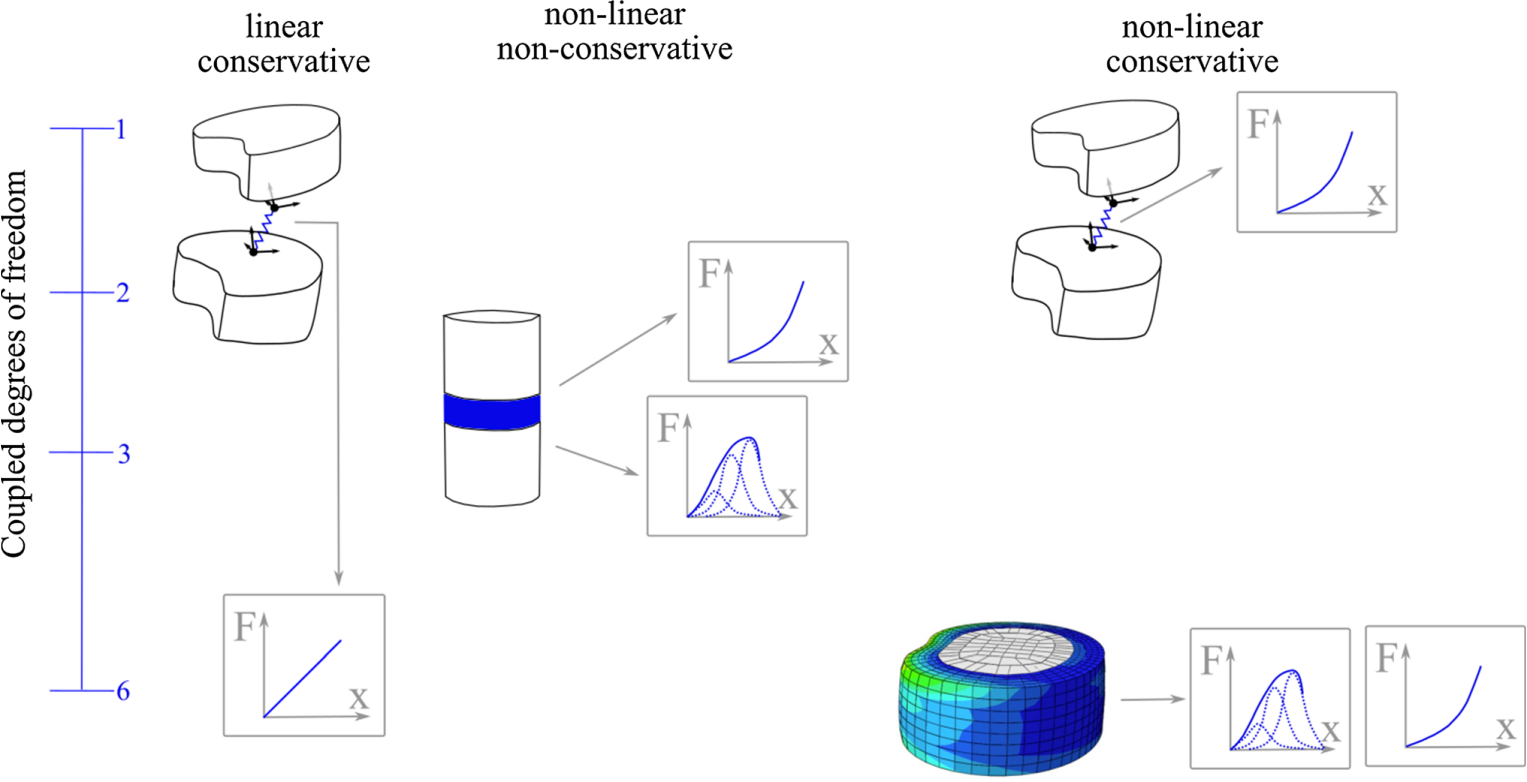
Overview of current surrogate modelling approaches for the elastic IVD responses. Linear stiffness matrices (top left), as for example Christophy et al. (2013) and Huynh et al. (2010), conserve energy and principally allow for coupling of all six DOFs. However, they often consider only two coupled DOFs or, when implemented as diagonal matrix, no coupling (coupled DOFs = 1). Similarly, uncoupled nonlinear models (top right) were developed from reported data (Damm et al. 2020; Schmid et al. 2020). Previous nonlinear models that including coupling (middle) were either polynomial approximations (Zhang et al. 2020; Karajan et al. 2013) with two coupled DOFs or kernel models (Wirtz et al. 2015; Haasdonk et al. 2021) with three coupled DOFs but did not conserve mechanical energy during motion. The first nonlinear surrogates that include coupling of all six DOFs, and conservation of energy (bottom right) are presented in this work and base on kernel or polynomial approximations

Surrogate modelling is a computationally inexpensive way to model complex, nonlinear biostructures in neuro-musculoskeletal (NMS) simulations of the spine used to non-invasively study generic and subject-specific spinal load sharing amongst active and different passive tissue compartments in predictive simulations (Meszaros-Beller et al. 2023; Mörl et al. 2020; Rupp et al. 2015; Guo et al. 2021). Particularly, the intervertebral disc (IVD) is a critical component, providing support and allowing for movement in all six degrees of freedom (DOFs). However, modelling the multiphasic soft matter physics of an IVD as a sub-model in subject-specific NMS spine models is challenging, especially under the demand of reduced model complexity to map the six-dimensional (6d) force-displacement behaviour. Apart from few attempts to directly model the IVD by mechanical components like springs in a multibody (MB) environment (Gao et al. 2015), the vast majority of spine models rely on data-based IVD models (see Fig. 1 for an overview of current modelling approaches), which are typically implemented as a force-torque element acting on adjacent vertebrae, while depending on the relative translational and rotational displacements of each vertebra. Such models should generally meet the following requirements: first, physical validity, second, exhibit the nonlinear force-displacement relations that are revealed experimentally, e.g., in Berkson et al. (1979), Schultz et al. (1979) and Panjabi et al. (1994), third, account for the coupling of the various DOFs, which has been documented in a few studies such as Patwardhan et al. (2003) and McGlashen et al. (1987), and, fourth, have a high accuracy on the given data set.

Disregarding the nonlinear characteristics of this soft cartilaginous tissue, the elastic IVD responses are commonly estimated by (bi-)linear spring-damping elements (Stokes and Gardner-Morse 1995; Huynh et al. 2010; Christophy et al. 2012; Meng et al. 2015; Meszaros-Beller et al. 2023; Senteler et al. 2016; Stokes et al. 2002; Monteiro et al. 2011). Even though these so-called bushing elements do only allow for linear coupling of DOFs, they are currently the most sophisticated approach for modelling elastic IVD responses in MB simulation frameworks in terms of considering fundamental physical principles, specifically the energy-conserving nature inherent to the term ‘elastic’. This fact is surprising, since it has already been elucidated more than one decade ago that the conservation of mechanical energy can be incorporated in MB IVD surrogates by deriving all vectorial forces and torques from a single scalar force-torque potential (see Senan and O’Reilly (2009) and Metzger et al. (2010)). However, existing nonlinear models of IVDs do either neglect mutual influences of DOFs (Damm et al. 2020; Schmid et al. 2020), or include coupling of only two or three DOFs by fitting force-displacement data for single force or torque components separately, e.g., with polynomials (Karajan et al. 2013; Zhang et al. 2020) or kernel approximations (Wirtz et al. 2015). Due to the lack of a common force potential and, by that, a loss of physical information, the latter published models violate energy balance.

Obtaining multidirectional force-displacement relations experimentally is not trivial and data of coupled DOF stiffnesses are still rare. Consequently, data-based models that include nonlinear coupling can hardly be trained on pure experimental data at present. As an alternative, detailed finite element (FE) models, validated against multiple experimental findings, can be used to provide estimates for the nonlinear and coupled elastic responses (Schmidt et al. 2007).

Several detailed, multiphasic FE models exist (Weisse et al. 2012; Ehlers et al. 2008; Karajan 2012; Little et al. 2008), which are often scaled to a subject-specific anatomy and generally capable of computing realistic IVD forces even under large and multidirectional deformations. However, their steep computational costs stand in contrast to the required reduced force law in a MB spine model, and can therefore not directly be implemented in the NMS model. To link detailed FE and NMS MB models, one can make use of hybrid models (Azari et al. 2018; Khoddam-Khorasani et al. 2018), with only slightly reduced computational cost compared to the pure FE models. A popular alternative approach is to use a detailed model to generate a multidimensional database and find a mathematical description, i.e., a surrogate, to map the resulting 6d displacements in an intervertebral joint to the six elastic responses component-wise (Karajan et al. 2013; Wirtz et al. 2015; Haasdonk et al. 2021). However, this procedure is associated with the aforementioned problem of the lack of energy conservation when kinetic coupling is included.

Consequently, the use of an FE IVD model developed to simulate a loading scenario similar to the utility of the NMS MB model, is suitable for generating a training data set. So, in this work, we employ a validated FE model from Little et al. (2008) for the subject-specific geometry of the Visible Male (Spitzer et al. 1996) and record its force-displacement relations. As surrogate models learn to map the model inputs to the desired outputs, and have no knowledge of the underlying physical system, there is a need to incorporate these physical laws directly into the model structure. Here, we modelled elastic response forces and torques, so we were aiming at surrogates that conserve the total energy. In mathematical terms, this is equivalent to requiring that the surrogate is the force field of an unknown potential. To enforce this condition, we define an IVD surrogate of a generic potential energy, and impose constraints on the value of its derivatives based on the measured force-displacement data of the detailed FE model. We apply this idea to define different types of surrogate models: polynomial models of low (second, third or fourth) order, and kernel models based on greedy kernel algorithms (Wendland 2005; Fasshauer and McCourt 2015; Wenzel et al. 2021). The novelty of this work lies in the design of these two IVD surrogate architectures that both combine the property of conservation of mechanical energy with existing fitting algorithms that consider the nonlinearity and kinetic coupling of the six DOFs of an intervertebral joint. Additionally, we quantified the accuracy of all surrogates to demonstrate the huge benefit of using nonlinear IVD models over common stiffness matrices in the context of predictive NMS MB simulations.

## Energy-conserving surrogate models

A surrogate model itself is no biophysical model in the sense of providing a full understanding of the mechanical responses exerted by microscopic structures. It is rather an approximation of the macroscopic output as a function of input variables. Therefore, desired features need to be anchored in the intrinsic model structure. As we aim for a surrogate model for the elastic response forces and torques, the model must not violate basic physical principles. In particular, it should not produce or annihilate energy during motion (no gain nor loss) because this would contradict the assumption of a purely elastic behaviour. To allow for kinetic coupling, the elastic responses that are exerted onto the adjacent vertebra endplates need to be treated as force-torque pairs rather than as uncoupled vector components.

In Sect. 2.1, we will recall the prerequisites of biomechanical surrogate models that map the 6d inputs of joint displacements to the 6d outputs comprising force and torque and at the same time ensure energy conservation from Senan and O’Reilly (2009) and Metzger et al. (2010) as the latter is regularly not taken into account in current IVD models. Afterwards, we give concrete classes of models, namely polynomial models (Sect. 2.2) and kernel models (Sect. 2.3) to realise the theoretical approach, and represent the force-torque pair by nonlinear, composite functions of coupled inputs. These models will be used and compared in the numerical simulations of Appendix D.

For readers who like to skip the math, the Sects. 2.1–2.3 can be summarised as follows: When a surrogate for the potential energy stored in an IVD is identified, instead of independently mapping each force and torque component, the model’s mechanical responses are automatically energy conserving. Thus, for an accurate modelling process, it is crucial to derive force and torque components from the first derivatives of the potential energy.

In the following, we use bold letters to denote vector valued quantities, i.e., $\varvec{x}_i$ for $i=1, \dots , N$ correspond to vector valued (input) data points. In contrast, non-bold letters correspond to scalar valued quantities, i.e., $x_\mu$ for $\mu =1, \dots , d$ correspond to the component of a vector $\varvec{x}$.

### Conservative force-torque field

The total mechanical energy conservation is a crucial feature of every elastic surrogate model, i.e., kinetic energy and potential energy may vary but their sum will stay constant over time independent of the loads applied. This is equivalent to the demand of zero net work on closed paths, i.e., the independence of mechanical work of the chosen (displacement) path. In other words, we can state that the surrogate model should represent a conservative force field (Senan and O’Reilly 2009; Metzger et al. 2010).

To elaborate this in more detail, we consider the potential energy $U(\varvec{x})$ as a function of the translational $\varvec{r}=(x_1,x_2,x_3)$ and rotational displacements $\varvec{\Phi }=(x_4,x_5,x_6)$, which together form the 6d state vector $\varvec{x}=(\varvec{r},\varvec{\Phi })$ of the intervertebral joint. Analogously, we define a vector of all six elastic IVD responses $\varvec{{\mathcal {F}}} =(\varvec{F},\varvec{M})$, containing three force components $\varvec{F}=({\mathcal {F}}_1,{\mathcal {F}}_2,{\mathcal {F}}_3)$ and three torque components $\varvec{M}=({\mathcal {F}}_4,{\mathcal {F}}_5,{\mathcal {F}}_6)$.

The existence of such a path- and time-independent potential *U* is mandatory for a conservative force field. According to the definition of a conservative force, the response forces and torques conserve mechanical energy if they can be derived from *U* as negative gradients

1 $$\begin{aligned} \nabla U = -{\mathcal {F}}^\prime \quad , \end{aligned}$$

with $\nabla =(\partial _{x_1}, \partial _{x_2}, \partial _{x_3}, \partial _{x_4}, \partial _{x_5}, \partial _{x_6})$ and $\partial _{x_\mu } \equiv \frac{\partial }{\partial x_\mu }$ for $\mu =1,2,...,6$, and where ${\mathcal {F}}^\prime$ represents the torque components in a different coordinate system, which can be easily converted in Cartesian coordinates^1^ to obtain $\mathcal F$.

To be called conservative, a force field ${\mathcal {F}}$ needs to be curl-free, i.e.,

2 $$\begin{aligned} \partial _{x_\mu } {\mathcal {F}}^\prime _\nu = \partial _{x_\nu } {\mathcal {F}}^\prime _\mu \quad \forall \ 1\le \nu ,\mu \le 6. \end{aligned}$$

We remark that curl-free is only a notation motivated by the case of ${\mathbb {R}}^3$, for which conservative vector fields are curl-free as the curl is zero. As the notation curl-free has been established in the literature (see e.g., Drake et al. (2021) and Drake et al. (2022)) also beyond the case of ${\mathbb {R}}^3$, we stick to it here.

Before proceeding with our formulation, we observe that this requirement is obviously fulfilled if the model neglects coupling between DOFs, i.e., if ${\mathcal {F}}^\prime _\mu ={\mathcal {F}}^\prime _\mu (x_\mu )$, as holds for, e.g., Schmid et al. (2020) and Mörl et al. (2020), or for symmetric stiffness matrices (Kövecses and Angeles 2007). On the other hand, the condition of Eq. (2) is automatically satisfied with the existence of a pot ntial *U* satisfying Eq. (1), as we can then rewrite Eq. (2) as $-\partial _{x_\nu }\partial _{x_\mu } U = -\partial _{x_\mu }\partial _{x_\nu } U$. The key to developing mechanical energy-conserving surrogates is, thus, to create a model for the force potential *U* and, afterwards, derive single force and torque components from it. In the following subsections, we present two different realisations of these elastic surrogate models including a coupling of different DOFs: polynomial models (Sect. 2.2) and greedy kernel models (Sect. 2.3).

We remark that from the practical point of view, for the computation of both types of models, we applied common preprocessing steps to the data. These include especially data cleaning (removing doubled points, removing outliers) and data transformation (scaling of the input to $[-1, +1]$). As scaling of the inputs also affects the outputs due to their relation from Eq. (1), no independent scaling of the outputs was applied. Further, no dimensionality reduction was applied as all features are inherently important for the conservation of energy.

### Energy-conserving polynomial models

In this subsection, we briefly present well-known polynomial models and elaborate how to use them for energy-conserving modelling. For our numerical experiments, we will consider polynomial models of degree two, three and four. For these low degrees, we can write generic polynomials as

3 $$\begin{aligned} q_2(\varvec{x})&= \sum _{i=1}^{6} p_i x_i + \sum _{i,k=1}^{6} p_{ik} x_i x_k\quad ,\nonumber \\ q_3(\varvec{x})&= q_2(\varvec{x}) + \sum _{i,k,j=1}^{6} p_{ikj} x_i x_k x_j\quad ,\nonumber \\ q_4(\varvec{x})&= q_3(\varvec{x}) + \sum _{i,k,j,l=1}^{6} p_{ikjl} x_i x_k x_j x_l\quad , \end{aligned}$$

where $p_i, p_{ik}, p_{ikj}, p_{ikjl}$, $1\le i,j,k,l\le 6$, are the free parameters of the model. Here, we omit the 0-degree monomial as explained later.

In order to predict the vector valued force-torque pair, simply using vector valued coefficients to map the single vector components independently does not result in energy-conserving surrogates as elaborated in Sect. 2.1. Instead, we predict the underlying (unknown) potential $U(\varvec{x})$ from Eq. (1) by fitting its derivative values, i.e., determining the coefficients of the polynomial model from Eq. (3) such that $\nabla U(\varvec{x})~\approx ~\nabla q_\ell (\varvec{x})$ for some $\ell =2,3,4$. As the values of the potential are unknown, we use the derivative values—which are provided as response forces and torques in force-displacement data sets—for the approximation. As an example, for $\mu =1, \ldots , 6$ we use in the case of a fourth-order polynomial the equations

4 $$\begin{aligned} {\mathcal {F}}^\prime_\mu= & {} \;\;\;\;\;\;\;\;\;\;\;\;\;\;\;\;\;\;\;-\frac{\partial q_4(\varvec{x})}{\partial x_\mu } \nonumber \\\!\!\!\!\!\!\!\!\!\!\!\!= & {} -p_\mu -2 \sum _{i=1}^{6} p_{\mu i} x_i -3 \sum _{i,k=1}^{6} p_{\mu ik} x_i x_k\nonumber \\{} & {} \;\;\;\;\;\;\;\;+ 4 \sum _{i,k,j=1}^{6} p_{\mu ikj} x_i x_k x_j \quad . \end{aligned}$$

The prefactors 2, 3 and 4 were explicitly included as we exploit the symmetry of the tensors $p_{\mu i}, p_{\mu i k}$, such that we can reduce the number of independent parameters. Note that the coefficient $p_{\mu }$ in Eq. (4) can in general be nonzero when the IVD is pre-strained and therefore exerts forces in the reference position where all displacements are zero, i.e., $\varvec{x}=0$.

The surrogate is obtained from imposing Eq. (4) for $\mu =1, \ldots , 6$ and for all $\varvec{x}$ in the data set. This results in a linear equation system which is, however, not necessarily square in general, as the number of equations ($d \cdot N$) usually does not match the number of (independent) parameters for the polynomial model from Eq. (3). Thus, the linear equation system is solved in a least-square sense. The corresponding calculations were done in Python using *PyTorch* (Paszke et al. 2017).

We remark that the 0-degree monomial is omitted in Eq. (3) as they are cancelled by differentiation and do not appear in Eq. (4). This corresponds to the fact that a potential is only uniquely defined up to the addition of constant terms.

### Energy-conserving kernel models

In the following, we use kernel models within this theory of conservative force-torque field (Wendland 2005; Fasshauer and McCourt 2015). The reader is referred to Appendix A for background information and an introduction into kernel methods with a focus on sparse kernel models obtained by greedy strategies. In fact, kernel methods generalise the polynomial models from the previous subsection, as those can be obtained using the so-called polynomial kernels. Thus, this broader class of methods allows for more sophisticated and accurate mathematical models.

We use the Gaussian kernel $k(\varvec{x},\varvec{z}) \in {\mathbb {R}}$ (see Appendix A, Eq. (7)), which has been used to train surrogates of single components of the IVD responses before (Wirtz et al. 2015; Haasdonk et al. 2021). However, here we exactly ensure the energy conservation property of Eq. (2) by using a curl-free variant of the Gaussian kernel (Drake et al. 2022), that is a matrix-valued kernel given by

5 $$\begin{aligned} \varvec{k}_{\textrm{cf}}(\varvec{x}, \varvec{z}) :=&-\nabla _{\varvec{x}} \nabla _{\varvec{z}}^\top k(\varvec{x},\varvec{z}) \in {\mathbb {R}}^{6 \times 6}, \nonumber \\ \Rightarrow k_{\textrm{cf}}(\varvec{x}, \varvec{z})_{\mu \nu } =&-\partial _{x_\mu } \partial _{z_\nu } k(\varvec{x}, \varvec{z})\quad , \end{aligned}$$

for $\mu , \nu = 1,..., 6$. By choosing a dimensionality of $d=6$, we account for the six DOF in an intervertebral joint. Then, the force-torque vector can be represented by the sparse kernel interpolant (see Eq. (8) for comparison), ${\mathcal {F}}^\prime \approx \varvec{s}_n(\varvec{x})$, with vector-valued coefficients $\varvec{\alpha }_i \in {\mathbb {R}}^6$ for $i=1,..., n$ (where the number *n* of used training points is a small subset of the training data inputs $\{ \varvec{x}_i\}_{i=1}^N, n\ll N$)

6 $$\begin{aligned} \varvec{s}_n(\varvec{x}) = \sum _{i=1}^n \varvec{k}_{\textrm{cf}}(\varvec{x}, \varvec{x}_i) \varvec{\alpha }_i \end{aligned}$$

with a common potential energy of all interpolant components,

$$\begin{aligned} U_{\varvec{s}_n}(\varvec{x}) = \sum _{i=1}^n\sum _{j=1}^d \left( \nabla _{\varvec{z}} k(\varvec{x}, \varvec{z}=\varvec{x}_i) \right) _j (\varvec{\alpha }_i)_j\quad . \end{aligned}$$

The vectorial coefficients $\varvec{\alpha }_i$ can be determined from the interpolation conditions $\varvec{s}_n(\varvec{x}_i) = \varvec{y}_i$ (with *n* of the training data outputs $\{\varvec{y}_i\}_{i=1}^n$ assigned to the kernels at $\{\varvec{x}_i\}_{i=1}^n$).

Note that several selection criteria give rise to meaningful subsets $X_n \subset X_N$. In this paper, we use a $\gamma$-*stabilised greedy algorithm*, which is a modification of standard greedy algorithms to yield more stable approximants $\varvec{s}_n$, see Wenzel et al. (2021). An implementation of the stabilised greedy algorithms for various kernels with a recent extension to the curl-free Gaussian kernel mentioned above in arbitrary dimensions $d \in {\mathbb {N}}$ can be found in the energy-conserving Vectorial Kernel Orthogonal Greedy Algorithm (VKOGA) implementation^2^ (Santin and Haasdonk 2021; Wenzel et al. 2021).

For the actual calculation of the models for all IVDs, we apply this VKOGA algorithm to the preprocessed data with a stabilisation parameter of $\gamma =0.05$. In particular, we run a 5-fold cross-validation to search for the best hyperparameters, precisely the best kernel width parameter $\epsilon$. As a search grid we used $\{\epsilon \}~\in ~\{ 0.5, 0.7, 0.9, 1.2 \}$. The maximal expansion size $n_\textrm{max}$ of the model was set to $n_\textrm{max} = 500$.

## Results

Both approaches from Sect. 2.2 and Sect. 2.3 were applied in the context of a digital human spine model to join the advantages of FE simulations of single tissue models and muscle-driven MB simulations with forward-dynamic capabilities. At the example of a subject-specific anatomy, we built surrogate models of detailed FE IVD representations, and implemented these in a MB thoracolumbar spine model (for generation of artificial training and test data, see Appendix D).

To evaluate the performance and accuracy of the kernel and polynomial modeling approach, we juxtaposed the different surrogate predictions with respect to the reference values gained using the underlying detailed FE IVD model. We started with an analysis of the models’ accuracy on the training data, followed by random six-DOF test displacements and ending with typical whole spine motions. This validation process is conducted exemplary for the L4$\vert$5 IVD geometry but could analogously be transferred to any other intervertebral level.

Additionally, we provide a verification test for elastic surrogate models (exemplary on the new kernel approach) in Appendix F.

### Accuracy on training and six-DOF test data

All surrogate models were created using single, two- and random six-DOF force-displacement training data sets. Their performance, in terms of predicted force precision, will here be compared to the training data itself and an additional six-DOF test data set. The reader is referred to Appendix D.1 for details about the data generation. Model predictions are compared to the reference data from the detailed FE model for all polynomial and kernel surrogates of the L4$\vert$5 IVD with respect to the average relative errors of force and torque values in Table 1.

For all surrogates, we found comparable relative errors throughout the different parts of the training data set. As expected, the second-order polynomial model showed the largest errors of on average 23.3% for forces and 16.9% for torques, followed by the third-order polynomial with 16.3% for forces and 15.9% for torques. The errors decreased with higher levels of polynomial order down to around 12.2% for forces and 9.6% for torques predicted by the fourth-order polynomial model. The lowest relative errors of, on average, 5.8% for forces and 6.5% for torques occurred using the kernel model.

**Fig. 2:**
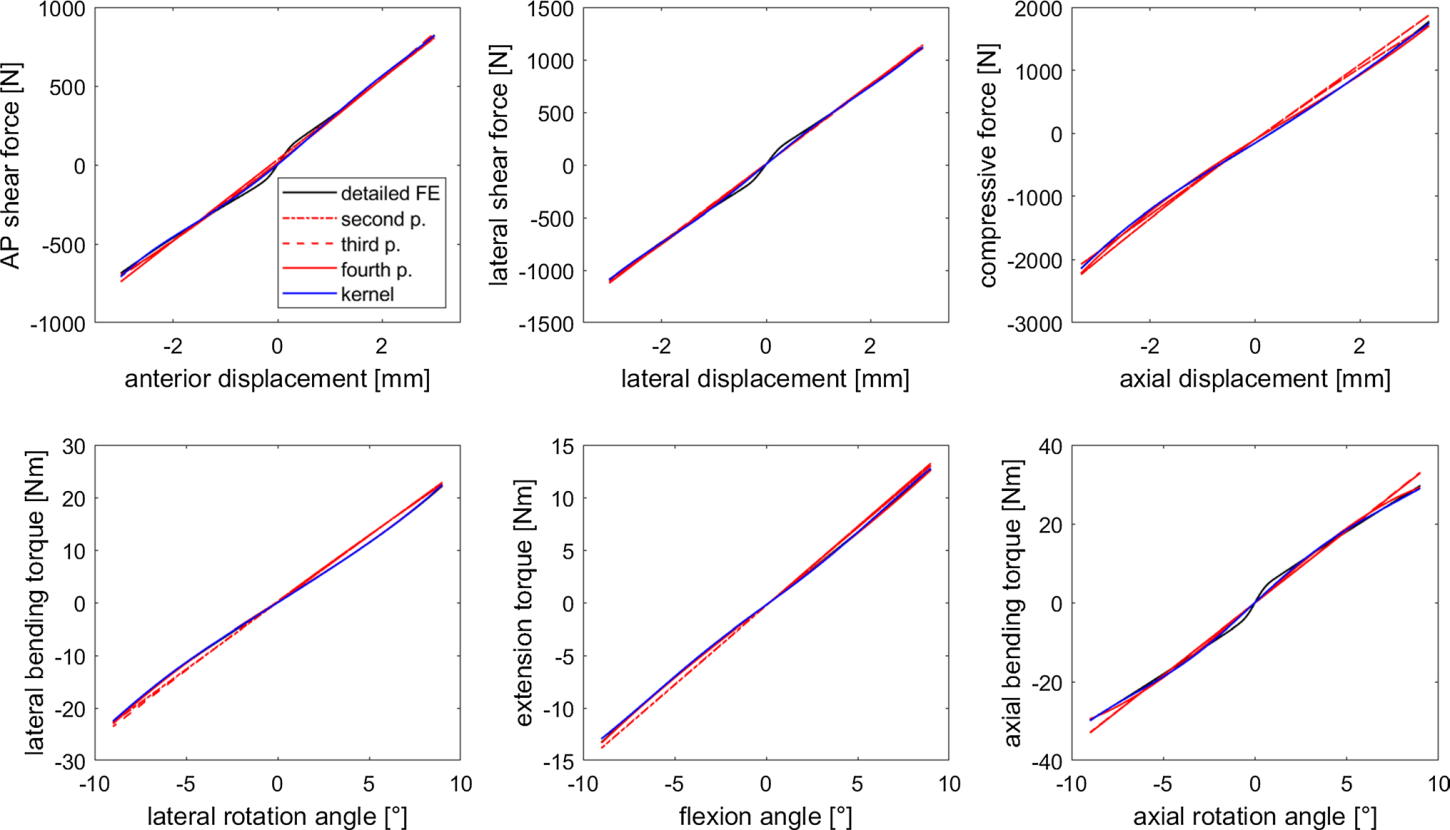
Comparison of single-DOF results for the detailed FE model of the L4$\vert$5 disc (black line) and the surrogates derived from it: kernel (blue line) and polynomial approximation (in red: second-order dotted line, third-order dashed line, fourth-order solid line). The six reaction force and torque components are displayed as a function associated with their corresponding displacement DOF and act in the opposite direction, i.e., anterior displacement results in a posteriorly (AP) directed shear force, lateral displacement or rotation to the right (negative lateral displacement values or positive lateral rotation angles) produces left lateral shear forces or bending moments, respectively, an axial compression (negative axial displacement) leads to tensile forces in the IVD, and a rotation about the caudo-cranial axis to a restoring force towards the relaxed position. Note that the pre-load of the IVD in the zero-displacement position causes an asymmetry in the compressive force values for positive and negative axial displacements

**Fig. 3:**
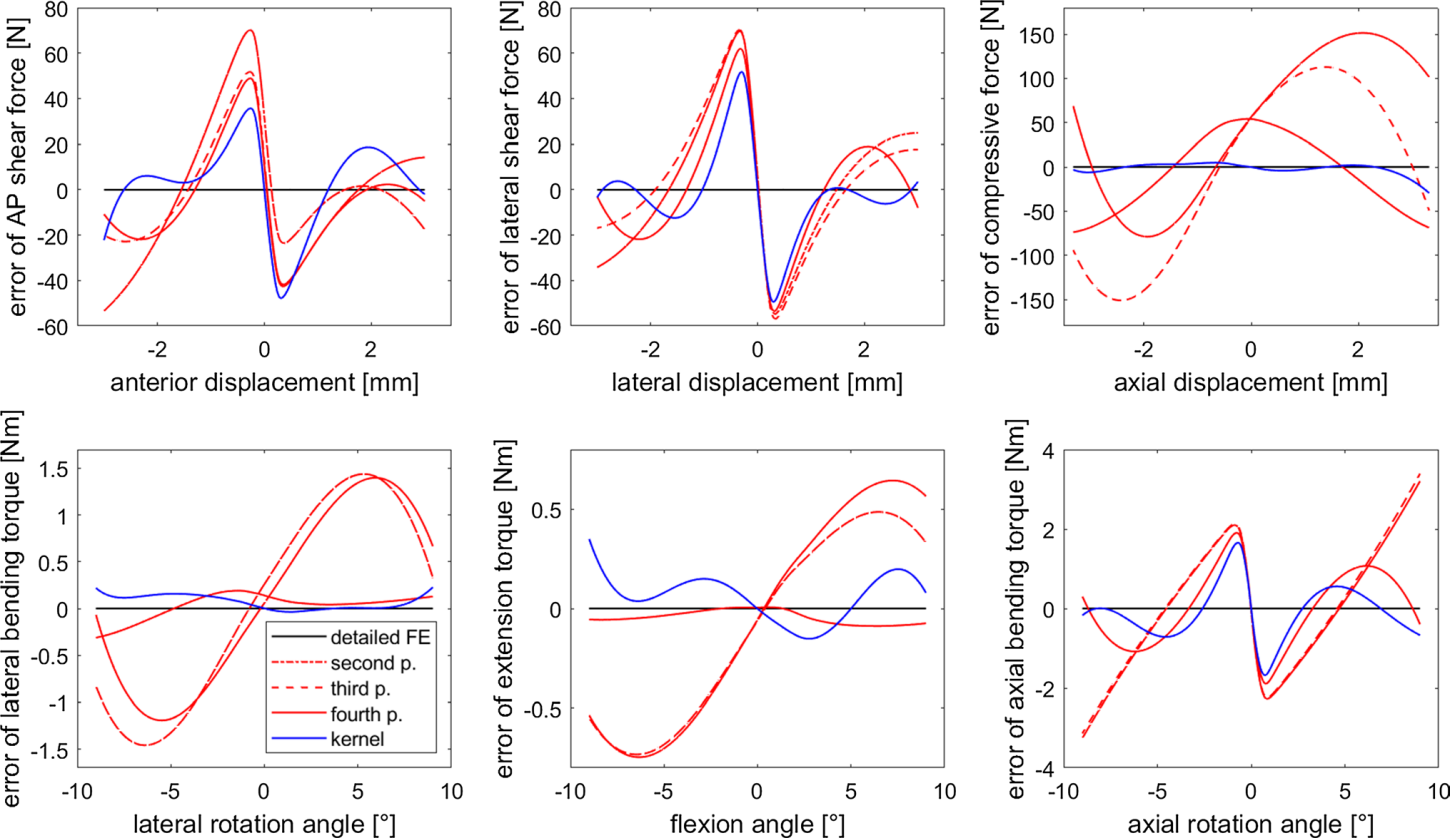
Deviations of the force and torque predictions of surrogate models from the reference data for single-DOF trajectories (compare Fig. 2)

A closer look at the single-DOF displacement trajectories, see Fig. 2, revealed that all models complied with the data and could be taken into consideration for use in biomechanical models. Only the deviations from the reference data, see Fig. 3, exhibited a clear difference between the IVD surrogate types. All models showed a similar pattern for lateral and anterior-posterior shear as well as for the axial rotation, whereas for lateral and frontal flexion and for compression, the fourth-order polynomial and kernel model were closer to the reference data, see Fig. 3.

Considering isolated force and torque components in the single-DOF simulations, we observed large relative errors in zero-crossing regions, i.e., when absolute values are small, across all surrogates and DOF. These errors decreased rapidly (but not monotonically) with larger excursions.

Polynomial models showed relative errors of 21.5%/ 20.1%, 15.7%/ 15.2% and 11.8%/ 10.5% for the forces/ torques predicted by the second-, third- and fourth-order polynomial on six-DOF test data. This was in the same range as the errors for the training data presented above. In contrast, for the kernel model, we saw a marked jump to 7.5% and 8.1% in the averaged force and torque errors, respectively. Thus, we presume that kernel models tend to be very precise in regions close to the training data but loose accuracy further away. This seems not to be the case for the polynomial models at the example of mapping 6d elastic IVD responses. However, judging from this set of training and random test data, the kernel model outperforms all polynomial models, especially on the full training data set but also markedly for the six-DOF test data where it still exhibits about 30% lower errors as compared to the most accurate polynomial model in this study.

**Table 1:** Average relative errors of predicted forces and torques (in $\%$) of the different IVD surrogate models (second-, third- and fourth-order polynomial and kernel approximation) with respect to the FE measurements for single-DOF (1D), two-DOF (2D) and random six-DOF (6D) force-displacement training data as well as unseen random six-DOF data which was not used for model training but as test data (6D test). The errors were computed as the norm of the respective absolute error vector normalised by the reference force or torque amplitude. In simulations with an asterisk, force and torque errors were calculated only for movement trajectories with nonzero translational or rotational displacements, respectively, in order to avoid domination of the zero-displacement state in the relative error estimation

		1D^∗^	2D^∗^	6D	6D test
Second p	Force	21.2	26.4	22.2	21.5
	Torque	13.2	17.3	20.3	20.1
Third p	Force	15.5	17.5	15.8	15.7
	Torque	16.5	16.2	15.1	15.2
Fourth p	Force	11.6	13.1	11.8	11.8
	Torque	8.7	9.7	10.3	10.5
Kernel	Force	4.8	6.6	5.9	7.5
	Torque	6.3	6.7	6.4	8.1

### Accuracy for spinal bending movements

In the second part of the model analysis, we investigated the IVD model precision on specifically relevant movement trajectories for biomechanics exemplary for three bending motions: forward bending, lateral bending to the right, and lateral bending to the left. For this, we created artificial kinematic data for the entire spine stemming from MB simulations of the subject-specific spine with kernel surrogates included, see Appendix D.2. The detected force-displacement data were not only applied to the detailed model to gain reference values but also to the three polynomial models to facilitate a comparison of the surrogate models’ accuracy on the same kinematic data.

**Fig. 4:**
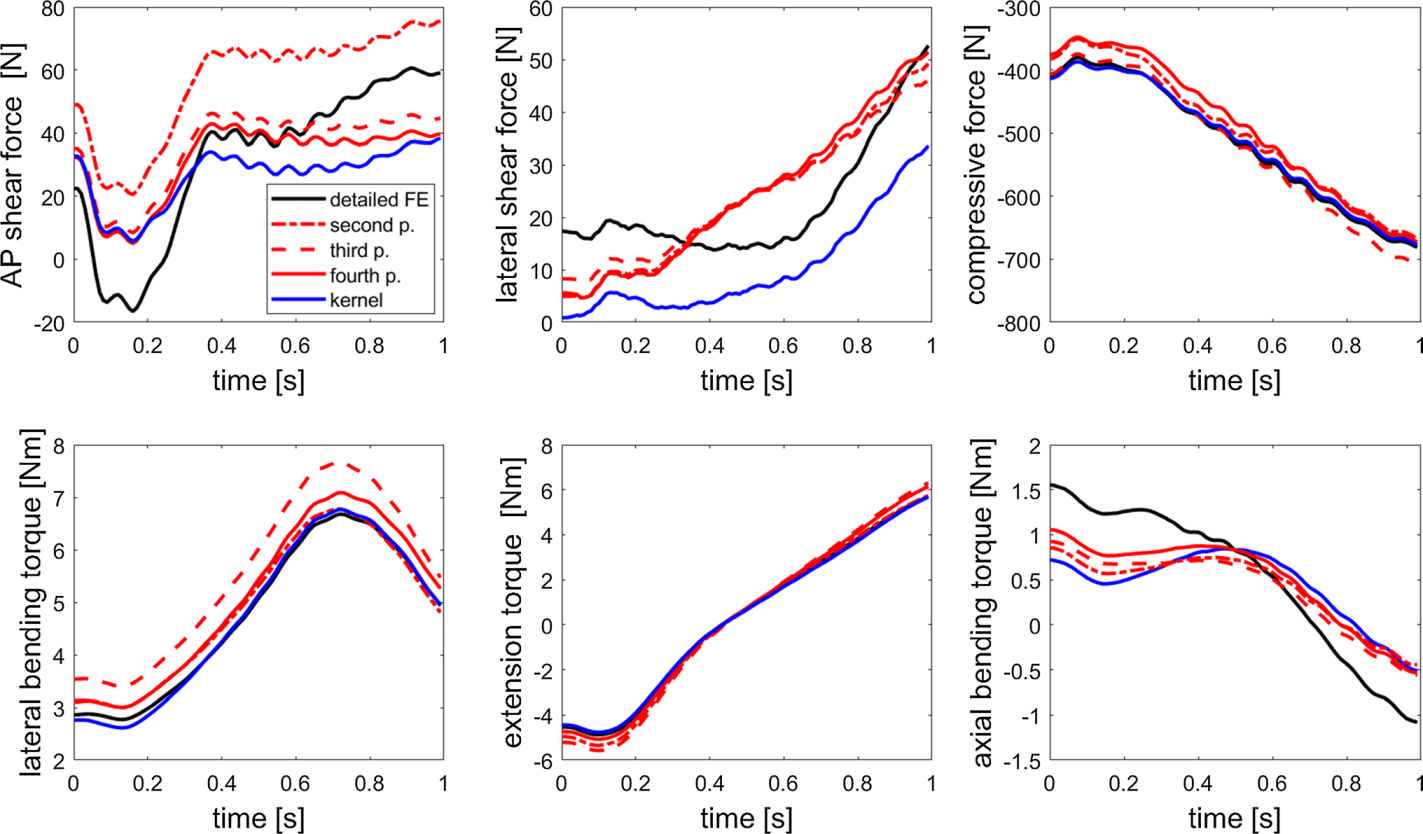
Kernel (blue line) and polynomial approximation predictions (in red: second-order dotted line, third-order dashed line, fourth-order solid line) of the three force and torque components for a forward bending movement trajectory in comparison with the reference data from the detailed FE model (black lines) for the same kinematics

**Fig. 6:**
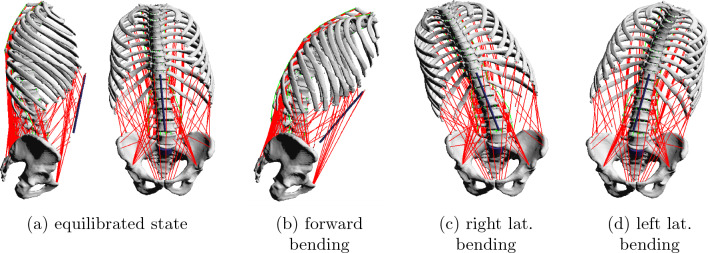
Forward (b) as well as right (c) and left (d) lateral bending simulations of the thoracolumbar spine model started from an equilibrated state (a) under gravitational loads

**Fig. 9:**
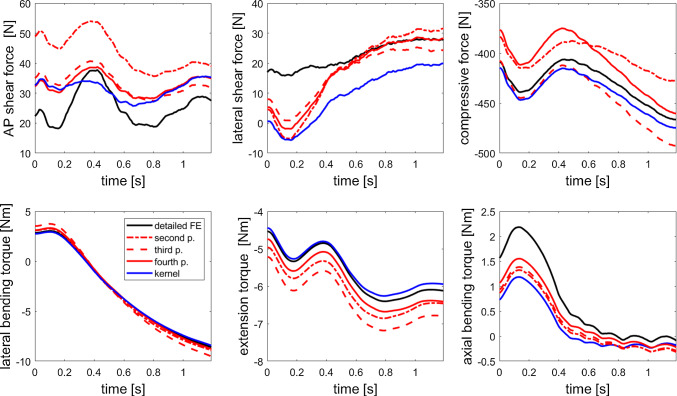
Kernel (blue line) and polynomial approximation predictions (in red: second-order dotted line, third-order dashed line, fourth-order solid line) of the three force and torque components for a **lateral bending movement to the left** in comparison with the reference data from the detailed FE model (black lines) for the same kinematics

**Fig. 10:**
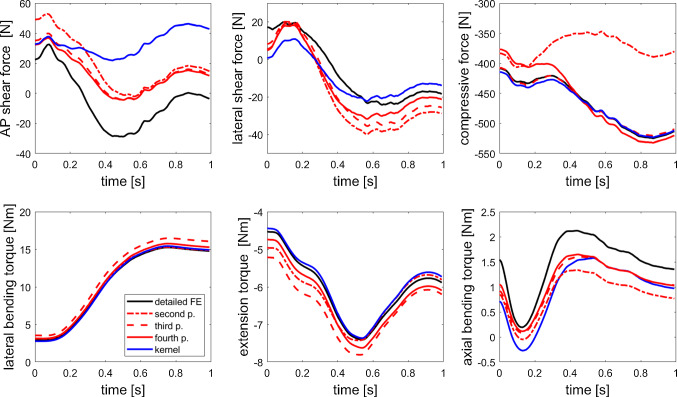
Kernel (blue line) and polynomial approximation predictions (in red: second-order dotted line, third-order dashed line, fourth-order solid line) of the three force and torque components for a **lateral bending movement to the right** in comparison with the reference data from the detailed FE model (black lines) for the same kinematics

For the forward and lateral bending motions, see Fig. 6, we observed a different model behaviour as seen before in Sect. 3.1. The performance of the surrogate models is depicted in Figs. 4, 9 and 10. First of all, the absolute values of forces and bending torques are lower as compared to *in vivo* measurements since head, neck and arm masses are neglected in the underlying NMS thoracolumbar spine model. However, this does not affect the surrogate model itself. Second, the relative errors were—on average—lower as compared to the six-DOF test data set, which can be attributed to the fact that there were no zero-crossings in the force and torque amplitude.

The mean relative errors varied a lot for the lower-order polynomials ranging from 6.6% to 20.7% for the second and 3.7% to 17.8% for the third-order polynomial with the largest errors occurring for the torque predictions. In contrast, the fourth-order polynomial and kernel model exhibited the most consistent relative errors of on average 6%/ 6.9% and 5.5%/ 7.4% for force/ torque error, respectively, see Table 2. An additional analysis of the errors of single force components and the measured elastic responses for the lateral bending can be found in Appendix G. Nevertheless, fourth-order polynomial and kernel approximation seem to be comparably precise in the spine bending test cases for the L4$\vert$5 IVD. Both outperform the commonly used linear stiffness matrix significantly.

**Table 2:** Average relative errors (in $\%$) of predicted forces and torques of all four surrogate models (second-, third- and fourth-order polynomial and kernel approximation) for typical spine movements: forward flexion (FF), left (LLB) and right lateral bending (RLB). Kinematic data were generated using an individualised MB spine model in combination with level-specific IVD kernel models

		FF	LLB	RLB
Second p	Force	7.0	7.8	20.7
	Torque	9.3	10.2	6.6
Third p	Force	3.7	4.2	4.4
	Torque	17.8	15.0	11.6
Fourth p	Force	6.8	5.6	5.5
	Torque	9.0	6.6	5.2
Kernel	Force	4.1	4.1	8.4
	Torque	8.5	8.2	5.5

## Discussion

To the best of our knowledge, this work is the first that combines the approach of creating a surrogate for a force potential with current sophisticated algorithms for nonlinear and high-dimensional force mapping in the context of biomechanics. By that, we present two novel surrogate architectures that intrinsically include the elastic nature of the IVD tissue: one based on kernel algorithms and one based on polynomial approximations. At the same time, we consider the large mutual impact of the different DOFs of an intervertebral joint onto their corresponding force and torque contributions. The resulting kernel and fourth-order polynomial models showed average relative errors of below 10% for all test and training data sets. Hence, when implemented in a MB simulation model, they can mimic the forces and torques of multiphasic, detailed IVD models onto the adjacent vertebral bodies with high precision compared to the second-order polynomial for the force potential. The latter corresponds, in fact, to the stiffness matrix of commonly used bushing elements. In this way, we are capable of simulating movements of the entire thoracolumbar spine at low computational costs. However, the usefulness of a surrogate depends largely on the appropriateness of its database. Alongside the quality of the different training and test data sets, we will discuss the biomechanical and methodological benefits and challenges of such surrogate models.

### Biomechanical quality of the training data and elastic modelling approach

While it would be valuable to develop the surrogate models using *in vitro* results for a cadaveric joint, whereby multi DOF loading is applied to a single IVD to provide multi-axis loading data for mathematical modelling, this is not feasible nor realistic due to specimen fatigue, creep and sub-failure micro-damage. As a basis for data acquisition, we therefore decided to use a detailed FE IVD model that has already been used in the clinical environment in the context of scoliosis surgery (Little et al. 2008; Little and Adam 2011). Validation of subject-specific osseoligamentous FE spine models including this IVD model has shown good agreement between experimentally measured stiffness and predicted stiffness for both joint level and overall spinal responses. Similarly, an analysis of the single-DOF force and torque predictions reveals a basic consistency with literature data (see Appendix E).

The generated surrogates shall serve as ideally elastic representations of the IVD responses. Since physiological IVD tissue has poroelastic as well as viscoelastic properties (Costi et al. 2008), the presented models can only approximate the IVD mechanics in a specific use case, i.e., under physiological loading conditions and strain rates. Consequently, the underlying FE model uses material parameters which are stiffer as compared to quasi-static experimental setups (e.g., from Berkson et al. (1979) and Schultz et al. (1979)), where creep-effects reduce the measured forces and torques. Additionally, a physiological pre-load increases linearity of force-displacement relations (Gardner-Morse and Stokes 2003; Zhang et al. 2020) which are nonlinear when starting in a relaxed state. Future representations of the IVD may include velocity dependent effects on stiffness and damping behaviour.

In this work, IVD surrogates were developed for the intervertebral level-, and subject-specific IVD mechanics for the anatomy of the VM (Spitzer et al. 1996)). Although it is known that the elastic responses of an IVD vary considerably interpersonally and with degeneration, these observations have rarely been quantified experimentally. Thus, subject-specific geometric differences, such as IVD conicity, are neglected in most MB spine simulation models, as there exists no scaling law for IVD forces on the adjacent vertebra endplates. Moreover, there is currently no non-invasive approach to prescribe mechanical soft tissue properties of subject-specific intervertebral discs. These facts restrict the IVD representations in NMS MB spine models to population-based and overly simplified models. Integrating surrogates of detailed, individualised IVD models into NMS models can partly overcome this challenge. We expect the level- and subject-specific IVD characteristics to substantially affect the load distribution determined in spine simulations as compared to population-based stiffness matrices averaged over several spinal levels as in Meszaros-Beller et al. (2023).

In our approach, mechanical responses of the detailed IVD model only scale with the subject-specific geometry while keeping the tissue stiffness parameters constant—disregarding possible gender, ageing, or traumatic effects. Ideally, multidimensional force-displacement data comprising material parameters derived from healthy and degenerated tissue would be used to set up a parametrised model that can be adapted to the subject’s conditions. This is, however, not a limitation of the general methodology presented in this work.

The kinematics used for the comparison between different IVD modelling approaches was gained from a predictive simulation of a NMS MB spine model. Even though the generic baseline model, from which the individualised model was derived, forecasted internal loads that were in good agreement with existing data on IVD, muscle and ligament forces during forward bending, the resulting movement trajectory might differ from the subject’s actual kinematics for the same bending task. A different joint angle trajectory could possibly lead to areas where models perform differently, i.e., with higher or lower average relative errors. However, we do not anticipate any fundamental changes in our conclusions when applying other displacements to the models.

### Challenges and benefits of the new methodology

For some detailed IVD models, it was not possible to collect data in the desired range of motion due to convergence issues of the FE numerics in the ABAQUS 6.14 solver. This was especially the case for very thin or conically shaped geometries and is expected to generally occur for degenerated thoracic and pathological IVDs. The different surrogate models for these IVDs were then trained with the same amount of data points but on a smaller displacement space. We initially intended to also model the L5$\vert$S1 IVD but since the FE simulations were restricted to a range below the compression levels reached during gravitational settling process, the MB simulations failed when including a L5$\vert$S1 kernel surrogate model into the individualised spine. Instead, we kept the bushing element for L5$\vert$S1 from Meszaros-Beller et al. (2023).

As described in Appendix D.1, we recorded data along trajectories starting from and returning to the starting point of zero displacements—therewith following a similar protocol as possible experimental data sets on coupled DOFs in the future. Thus, we find a data-rich area around this point. In contrast, only 200 displacement trajectories were used to detect forces in the entire 6d space resulting in data-sparse areas for the highly coupled DOF motions and specifically at the boundaries. The bending simulations, however, started from an equilibrated position under gravitational loads which is not equivalent to the zero-displacement position referred to as the initial state. This explains the slightly larger deviations from the detailed model at the beginning of the movement simulations (see Figs. 4, 9 and 10) as compared to the purely one-dimensional displacement predictions (Fig. 3). A more systematic scan of the 6d space enclosed by the physiological boundaries defined in Appendix D.1, or further data points would presumably improve model performances for unknown trajectories such as the spine bending motions. In particular, the kernel approximation could profit as it shows the only marked jump in relative errors from training to unseen test data probably due to the inhomogeneously distributed locations of the kernel centres.

We observed a systematic decrease in relative errors for an increasing polynomial order of the surrogates. However, the underlying algorithm uses tensor multiplications with tensors of the same order as the polynomial. This complicates the model development when adding terms of higher order to capture the high-dimensional coupling of DOFs. In contrast, kernel approximations can easily handle functions with high-dimensional inputs with calculations being based on common matrix multiplications.

The higher accuracy and improved practicality for 6d fitting comes with an increasing number of model parameters. While the second-order polynomial has only 21 independent values in the stiffness matrix and the 6 forces and torques in the zero-displacement state (in total 27), the third-order polynomial requires additional 56 parameters for the symmetric third-order tensor (83 in total), and a fourth-order polynomial needs additional 126 parameters to describe the symmetric fourth-order tensor (summing up to 209 independent parameters). The actual number of kernel surrogate parameters varies depending on the number of kernels used by the algorithm, which was limited to a maximum 500. For the L4$\vert$5 kernel model, this was 399 kernels with 6 parameters for the corresponding vectorial weighting factor plus one for the kernel width (in total 2,394 independent model parameters). In the current work, the focus laid on including a given data set—here, the finite element model mechanics—into a multibody environment with the highest possible precision. Thus, number of model parameters played a minor role. However, a larger model complexity might cause higher computational cost, or be relevant for certain applications. This effort is—for both the polynomial and kernel model—orders of magnitude lower than the underlying finite element model, and reasonably small in our tests.

While kernel models perform excellently on the range of motion defined by the boundaries of the data set (see Appendix D.1) and particularly on the training data, a limitation of the kernel surrogate models is its limited generalisation performance to unseen physical regimes of the data: the used Gaussian kernel is a localised function, and as all the recorded data lies in a bounded 6d box, the kernel surrogate model can only be expected to perform well within this box. Outside the boundaries, its performance will likely deteriorate and approach zero for larger excursions. This can be seen as a benefit of the polynomial models, as they actually can suitably predict growing forces and torques for increasing displacements, even outside the box of collected data points. A future extension of this project would be to combine kernel and polynomial approaches in order to benefit from the extrapolation capabilities of polynomial models and, at the same time, from the precision of kernel models especially in data-rich regions.

Also, it seems worth investigating further strategies to improve the accuracy of the surrogates. Higher-order polynomials or different kernel types, e.g., polynomial kernels, might be an alternative choice for the mostly monotonous force-displacement curve.

In Appendix F, we present a test to verify the elastic soft-tissue models with respect to energy conservation. The results demonstrate that surrogates developed here do, indeed, conserve energy—in contrast to previously presented approaches such as Haasdonk et al. (2021). This analysis also shows that although the training data were gained with an ideally elastic FE IVD model, the surrogates with independently fitted output forces and torques are not fully elastic. Even if computed net work was low for the non-conserving force in the chosen example, errors can add up over time particularly when several of such energy sinks or sources are implemented. This highlights the necessity to incorporate fundamental physical principles in the surrogate development process.

## Conclusion

With the presented work, we demonstrate a way to design and develop surrogate models for multidimensional data sets of the elastic responses of soft tissues such as IVDs. By deriving all force and torque components from one single force potential, we ensure the conservation of mechanical energy in the modelled material. In combination with greedy kernel and polynomial approximations of detailed FE IVD models, we gained precise models capable of predicting the nonlinear and coupled force-displacement behaviour of IVDs in a MB spine simulation. Using this approach, it is also possible to increase the level of individualisation in subject-specific MB spine models with respect to level- and subject-specific IVD characteristics.

## Figures and Tables

**Fig. 5:**
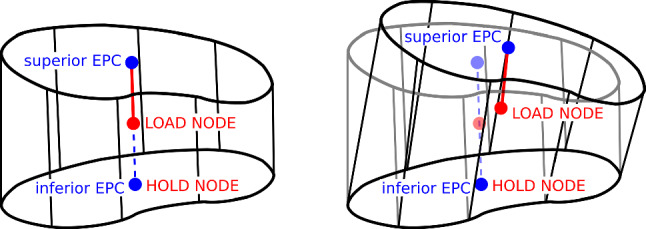
Definition of specific nodes in the level-specific detailed IVD models. The inferior endplate was kept fixed over time by placing the *HOLD NODE* at the inferior EPC. Displacement loads were applied to the superior endplate by a rigid beam connector the *LOAD NODE*, which was positioned at the arithmetic mean of both adjacent EPCs in the initial state

**Fig. 7:**
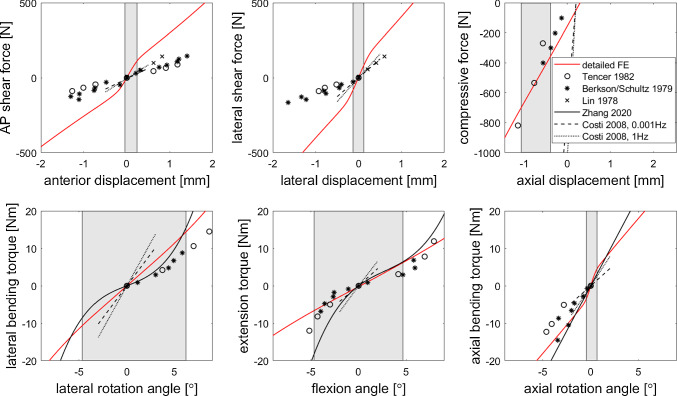
Comparing elastic responses of a subject-specific detailed FE model of the L4$\vert$5 disc (red line) against experimental data from literature (black circles, crosses and asterisks) (Tencer et al. 1982; Berkson et al. 1979; Schultz et al. 1979; Lin et al. 1978), linear regressions to velocity dependent disc responses for two different movement frequencies in the plotted displacement range (Costi et al. 2008), and a recent meta-regression analysis (solid black line) (Zhang et al. 2020). The intervertebral joint displacements occurring during the spinal bending motions described in Appendix D.2 are indicated by grey shaded areas. Note that we assumed a constant compressive loading of 155 N for the regression fit of a L4$\vert$5 disc. This value equals the compressive force acting in the zero-displacement state of the detailed model

**Fig. 8:**
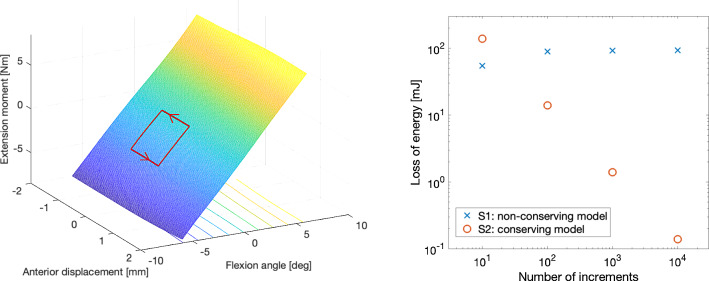
The energy loss or gain is calculated by integration of the force-torque vector over the joint displacement vector along a closed movement trajectory (red line on the left). It is quantified in terms of the net power (right plot) for non-conservative (S1, blue crosses) and conservative models (S2, red circles) depending on the discretisation, i.e., the number of increments on every path

## Appendix A: Kernel methods

In the following, we briefly introduce kernel methods (Wendland 2005; Fasshauer and McCourt 2015) with a focus on sparse greedy kernel models. The notion *kernel methods* comprises useful machine learning techniques and high-dimensional scattered data approximation. These methods work solely based on the data and are especially independent of the data distribution.

The key ingredient is a kernel *k*, which is a symmetric function $k: \Omega \times \Omega \rightarrow {\mathbb {R}}$ on a given domain $\Omega$. Here, we will use $\Omega \subset {\mathbb {R}}^d$ with $d=6$, although kernels are generally applicable in general space dimensions $d \in {\mathbb {N}}$. A well-known kernel is the Gaussian kernel, which is given by

7 $$\begin{aligned} k(\varvec{x}, \varvec{z}) = e^{- \epsilon ^2 \Vert \varvec{x} - \varvec{z} \Vert _2^2} \in {\mathbb {R}}, \;\;\varvec{x}, \varvec{z} \in {\mathbb {R}}^d, \end{aligned}$$

with a kernel width parameter $\epsilon > 0$. Associated with a kernel and a data set $(X_N, Y_N) = (\{ \varvec{x}_i \}_{i=1}^N, \{ y_i \}_{i=1}^N ) \subset (\Omega \times {\mathbb {R}})^N$, we define the kernel matrix $\varvec{K}_{X_N}$ as

$$\begin{aligned} (K_{X_N})_{ij} = k(\varvec{x}_i, \varvec{x}_j), \qquad 1 \le i, j \le N. \end{aligned}$$

Only if this kernel matrix $\varvec{K}_{X_N}$ is strictly positive definite for any choice of pairwise distinct data points $\{ \varvec{x}_i \}_{i=1}^N$, the kernel is called positive definite. This is the case for the Gaussian kernel. In this case, the mathematics gives rise to an associated space of functions, the so-called Reproducing Kernel Hilbert Space (RKHS) ${\mathcal {H}}_k(\Omega )$. Inside this space of functions, a unique interpolating function $s_N$ to the data set $(X_N, Y_N)$ can be found. In particular, a representer theorem (Kimeldorf and Wahba 1970; Schölkopf et al. 2001) states that this interpolant $s_N$ can be expressed as

8 $$\begin{aligned} s_N(\varvec{x}) = \sum _{i=1}^N \alpha _i k(\varvec{x}, \varvec{x}_i) \quad , \end{aligned}$$

where the coefficients $\varvec{\alpha } = (\alpha _i)_{i=1}^N \in {\mathbb {R}}^N$ can be calculated with help of the kernel matrix $\varvec{K}_{X_N}$ and the target value vector $\varvec{y} = (y_i)_{i=1}^N$ by solving the linear equation system

9 $$\begin{aligned} \varvec{K}_{X_N} \varvec{\alpha } = \varvec{y}, \end{aligned}$$

which arises from the interpolation conditions $s_N(\varvec{x}_i) = y_i$ for all $i=1,.., N$. Provable error estimates show a rapid and precise approximation with few expansion points (Wendland 2005; Santin and Haasdonk 2017; Wenzel et al. 2023).

The same holds for the here applied curl-free Gaussian kernel (see Sect. 2.3, Eq. (5)), where the kernel interpolant takes a similar form as in Eq. (8), though with vector-valued coefficients $\varvec{\alpha }_i$ (see Eq. (6)). Again, the coefficients $\varvec{\alpha }_i$ can be determined from the interpolation conditions $\varvec{s}_N(\varvec{x}_i) = \varvec{y}_i$, which leads to a linear system of size $dN \times dN$ similar to Eq. (9).

The computation of the kernel matrix $\varvec{K}_{X_N}$ and a straightforward solving for the coefficient vectors $\varvec{\alpha }_i$ of the equation system Eq. (9) have a cost of order ${\mathcal {O}}(d^2N^2)$ and ${\mathcal {O}}(d^3N^3)$, respectively. This is prohibitively expensive for large data sets, as in the case of the simulation of the IVDs (see Appendix D). Thus, we employ greedy algorithms that result in sparse interpolants $\varvec{s}_n$ with $n \ll N$ by selecting a proper subset $X_n \subset X_N$ of the training data. These algorithms start with an empty interpolation set $X_0:= \emptyset$ and iteratively add another interpolation point as $X_{n+1}:= X_n \cup \{\varvec{x}_{n+1}\}$ according to a selection criterion $\eta ^{(n)}$, i.e.,

$$\begin{aligned} \varvec{x}_{n+1} := \textrm{arg max}_{\varvec{x} \in \Omega } \eta ^{(n)}(\varvec{x}). \end{aligned}$$

A variety of selection criteria allows to balance between, e.g., accuracy and stability of the resulting model, see (Wenzel et al. 2021, 2023).

## Appendix B: Definition of joint reference frames

In the current study, we adapted the joint body configuration and the VB and joint frame definition of the individualised model from Meszaros-Beller et al. (2023) (Appendices 1-3). Accordingly, joint reference frames ${\mathcal {O}}_\text {JNT}^{i}$ (*i* denotes the intervertebral level from L5$\vert$S1 to T1$\vert$2 when enumerating joints) were chosen to be located at the arithmetic mean of the geometric centres of sub- and superjacent VB endplates, the EPCs, and their orientation set to the average orientation of both adjacent endplates. Two corresponding joint reference frames of each intervertebral joint, one fixed to the parent and the other fixed to the child body, coincide in the initial position. Thus, the location of the joint reference frames between their superjacent VB with inferior EPC at $\varvec{P}_\text {EPC}^{\text {inf},i+1}$ and its subjacent VB with superior EPC at $\varvec{P}_\text {EPC}^{\text {sup},i}$ in the initial position is given by

$$\begin{aligned} \varvec{P}_{\text {JNT},i}=(\varvec{P}_\text {EPC}^{\text {inf},i+1}+\varvec{P}_\text {EPC}^{\text {sup},i})/2 \quad , \end{aligned}$$

where *i* ranges from sacrum (S1) to the first thoracic (T1) for VBs and respective EPCs.

When determining the orientation of ${\mathcal {O}}_\text {JNT}^{i}$, we calculate the average orientation of the coordinate systems of both adjacent endplates, ${\mathcal {O}}_\text {EPC}^{\text {inf},i+1}$ and ${\mathcal {O}}_\text {EPC}^{\text {sup},i}$, such that the rotation matrix from ${\mathcal {O}}_\text {EPC}^{\text {sup},i}$ to ${\mathcal {O}}_\text {JNT}^{i}$ is exactly the inverse matrix to describe the rotation from ${\mathcal {O}}_\text {EPC}^{\text {inf},i+1}$ to ${\mathcal {O}}_\text {JNT}^{i}$. In general, a rotation matrix can fully be parameterised by three mutually independent quantities. These quantities may be quaternions or axis-angle representations with two independent quantities for the normalised rotation axis and one for the rotation magnitude. Alternatively, a set of three angles is used which can be of Cardan or Euler type, a rotation vector—related to axis-angles—or, with a deeper biomechanical interpretation, anatomical angles (Pearcy and Tibrewal 1984) or projected angles onto specific planes, e.g., sagittal, coronal and horizontal plane. In contrast to most biomechanical modelling approaches of the IVD based on Euler or Cardan angles (Christophy et al. 2013; Karajan et al. 2013), we decided to use the angle-axis representation for the computation of joint orientations and the related rotation-vector notation for rotational displacements in the joint because of four aspects: First, the angle-axis and rotation-vector conventions have a unique solution when restricting to rotation magnitudes below $180^\circ$, which is an appropriate assumption the case in the context of the intervertebral joint. Second, these representations are biomechanically meaningful as the angle vector immediately gives insight into the rotation axis of the intervertebral joint and the overall magnitude of the rotation angle. Third, both are comparably simple to be transposed since there are no distinct mutual dependencies between the three angle components. This is especially relevant when transferring models from one simulation tool to the other, where the order of unit axis or, in case of Euler and Cardan angles, the order of consecutively executed rotations around the single unit axes might be different. Fourth, they are closely related to quaternions, which eases the transformation of torques derived from a force potential into Cartesian torque vectors (see Appendix C).

Using the angle-axis representation for the relative orientation of ${\mathcal {O}}_\text {EPC}^{\text {sup},i}$ with respect to ${\mathcal {O}}_\text {EPC}^{\text {inf},i+1}$, we find ${\mathcal {O}}_\text {JNT}^{i}$ by halving the rotation magnitude. The detailed procedure is presented in the following. We calculate the rotation axis $\varvec{t}$ and angle $\theta$ representing the rotation matrix from ${\mathcal {O}}_\text {EPC}^{\text {sup},i}$ to ${\mathcal {O}}_\text {EPC}^{\text {inf},i+1}$, $\varvec{R}_{\text {sup},i\rightarrow \text {inf},i+1} = (\varvec{R}_{\text {sup},i})^{-1}\cdot \varvec{R}_{\text {inf},i+1}$:

$$\begin{aligned} \theta= & {} \arccos \left( \frac{\textrm{Tr} (\varvec{R}_{\text {sup},i\rightarrow \text {inf},i+1})-1}{2}\right) \\ \varvec{T}= & {} \frac{\varvec{R}_{\text {sup},i\rightarrow \text {inf},i+1}-(\varvec{R}_{\text {sup},i\rightarrow \text {inf},i+1})^{-1}}{2\cdot \sin \theta } \quad . \end{aligned}$$

where Tr($\varvec{R}_{\text {sup},i\rightarrow \text {inf},i+1}$) is the matrix’ trace and $\varvec{T}$ the skew-symmetric matrix of the rotation axis $\varvec{t}=(t_x,t_y,t_z)$,

$$\begin{aligned} \varvec{T}= & {} \left( \begin{array}{c c c} 0 &{} -t_z &{} t_y\\ t_z&{} 0 &{} -t_x\\ -t_y &{} t_x &{} 0 \end{array}\right) \quad . \end{aligned}$$

Then, the orientation ${\mathcal {O}}_\text {JNT}^{i}$ relative to ${\mathcal {O}}_\text {EPC}^{\text {sup},i}$ is found by rotating about the same axis $\varvec{t}$ as above by $\theta /2$, and can be calculated following Rodrigues’ rotation formula:

$$\begin{aligned} \varvec{R}_{\text {sup},i\rightarrow \text {JNT},i}= & {} \varvec{I} + \sin \left( \frac{\theta }{2}\right) \cdot \varvec{T} \\{} & {} + \left( 1-\cos \left( \frac{\theta }{2}\right) \right) \cdot \left( \varvec{T}\right) ^2 \end{aligned}$$

with *I* denoting the identity matrix.

Afterwards, the rotation matrix $\varvec{R}_{\text {sup},i\rightarrow \text {JNT},i}$ given in local coordinates of the subjacent vertebral endplate is back-transformed into the global reference frame to receive a description of the average coordinate system orientation in the global reference frame, $\varvec{R}_{\text {JNT},i}=\varvec{R}_{\text {sup},i}\cdot \varvec{R}_{\text {sup},i\rightarrow \text {JNT},i}$.

Finally, we want to take the note that the same definition of joint position and orientation was used as reference frame when recording the force-displacement data, see Appendix D.1. We therefore defined the surrogate reference frames to coincide with the joint reference frames also in the MB simulations, see Appendix D.2.

## Appendix C Conservative momentum for rotation-vector angles

In order to create surrogate models for the force potential (the potential energy) of an IVD, and to correlate its derivatives to the forces and torques on the vertebra, the methodology used in Metzger et al. (2010), O’Reilly and Srinivasa (2002) and Senan and O’Reilly (2009) is followed. In contrast to these works, which are based on the general theory on moment potentials (Simmonds 1971, 1984) and its application to rotations parametrised by quaternions and Euler angles, we here elaborate the equations for the use of a rotation-vector notation as justified in Appendix B. That means that our force potential $U=U(\varvec{r},\varvec{\Phi })$ depends explicitly on the translational displacements $\varvec{r}$ in the intervertebral joint and on the rotation-vector $\varvec{\Phi }=(x_4,x_5,x_6)$ describing the rotational displacement. The rotation vector itself includes information about the magnitude $\Phi =\Vert \varvec{\Phi }\Vert$ of the rotation about the normalised axis $\varvec{u}=\varvec{\Phi }/\Phi$.

The change of potential energy when moving along a path is described by the following relation, where the time dependency of the force potential is defined by the work performed in this time against the translational displacement $\varvec{F}\cdot \dot{\varvec{r}}$ and the rotational displacement $\varvec{M}\cdot \varvec{\omega }$, i.e.,

10 $$\begin{aligned} -\dot{U}(\varvec{r},\varvec{\Phi }) = \varvec{F}\cdot \dot{\varvec{r}} + \varvec{M}\cdot {\varvec{\omega }} \quad , \end{aligned}$$

with translational velocity $\dot{\varvec{r}}$ and rotational velocity vector $\varvec{\omega }$. Without an explicit time dependency of *U*, we can rewrite the left hand side into

11 $$\begin{aligned} -\dot{U}(\varvec{r},\varvec{\Phi }) =-\frac{\partial U}{\partial \varvec{r}}\cdot \dot{\varvec{r}}-\frac{\partial U}{\partial \varvec{\Phi }}\cdot \dot{\varvec{\Phi }}\quad . \end{aligned}$$

From the first term, we identify $\varvec{F}=-\frac{\partial U}{\partial \varvec{r}}$. In order to calculate the torque $\varvec{M}$ from the gradient of the force potential $\varvec{M}^\prime =-\frac{\partial U}{\partial \varvec{\Phi }}$, we must re-formulate the second term. For this, we do a brief excursion developing $\dot{\varvec{\Phi }}$.

The time derivative of the angle vector can be accessed via the time derivative of the corresponding quaternion $\varvec{q}=(\cos (\Phi /2),\varvec{u}\cdot \sin (\Phi /2))$, which is expressed as quaternion product of the angular velocity and the quaternion itself:

12 $$\begin{aligned} \frac{\textrm{d}\varvec{q}}{\textrm{d}t}=\frac{1}{2}\left( \begin{matrix} 0 \\ \varvec{\omega }\end{matrix}\right) \varvec{q}. \end{aligned}$$

Reformulating left and right side of Eq. (12), we get

13 $$\begin{aligned}{} & {} \left( \begin{matrix} -\sin (\frac{\Phi }{2})\cdot \frac{\dot{\Phi }}{2}\\ \dot{\varvec{u}}\cdot \sin (\frac{\Phi }{2})+\varvec{u}\cdot \cos (\frac{\Phi }{2})\cdot \frac{\dot{\Phi }}{2}\end{matrix}\right) \nonumber \\{} & {} \quad =\frac{1}{2}\left( \begin{matrix} -\varvec{\omega }\cdot \varvec{u}\cdot \sin (\frac{\Phi }{2})\\ \varvec{\omega }\cdot \cos (\frac{\Phi }{2}) + \varvec{\omega } \times \varvec{u}\cdot \sin (\frac{\Phi }{2})\end{matrix}\right) . \end{aligned}$$

The scalar and vector components of Eq. (13) yield the two relations

14 $$\begin{aligned} \dot{\Phi }&= \varvec{\omega }\cdot \varvec{u} \end{aligned}$$

15 $$\begin{aligned} 2\cdot \dot{\varvec{u}}\sin (\frac{\Phi }{2})&= \varvec{\omega } \times \varvec{u}\cdot \sin (\frac{\Phi }{2}) \nonumber \\&+\left[ \varvec{\omega }-\varvec{u} (\varvec{\omega }\cdot \varvec{u})\right] \cdot \cos (\frac{\Phi }{2})\quad . \end{aligned}$$

Finally, with $\left[ \varvec{\omega }-\varvec{u} (\varvec{\omega }\cdot \varvec{u})\right] = \varvec{u}\times (\varvec{\omega }\times \varvec{u})$, we can derive

16 $$\begin{aligned} \dot{\varvec{\Phi }}= & {} \Phi \cdot \dot{\varvec{u}}+\dot{\Phi }\cdot \varvec{u} \end{aligned}$$

17 $$\begin{aligned}= & {} \frac{\Phi }{2} \left[ \varvec{\omega } \times \varvec{u} + \varvec{u}\times (\varvec{\omega }\times \varvec{u}) \cdot \cot \left( \frac{\Phi }{2}\right) \right] \nonumber \\{} & {} +\varvec{u}\cdot (\varvec{\omega }\cdot \varvec{u}) \end{aligned}$$

for all $\Phi \ne 0$. Note that $\varvec{u}$ is not defined if $\Phi =0$, and we set $\dot{\varvec{\Phi }}=\varvec{\omega }$ in this case.

Returning to Eq. (11), we rewrite the term that accounts for the implicit time dependency of *U* through the rotation vector and insert Eq. (17). Comparing the derived term with Eq. (10) leads us to a formulation of the torque vector $\varvec{M}$:

18 $$\begin{aligned} -\frac{\partial U}{\partial \varvec{\Phi }}\cdot \dot{\varvec{\Phi }}= & {} \varvec\;\;\;{M^\prime }\cdot \left[ \mathbf{{u}}\cdot (\varvec{\omega }\cdot \varvec{u})+\frac{\Phi }{2}\left(\varvec{\omega }\times \varvec{u}\right.\right. \nonumber \\{} & {} \left. +\varvec{u}\times \left.(\varvec{\omega }\times \varvec{u})\cdot \cot {\left(\frac{\Phi}{2}\right)}\right)\right] \nonumber \\= & {} \varvec{\omega }\cdot \left[ (\varvec{M}^\prime \cdot \varvec{u}) \cdot \varvec{u}+\frac{\Phi }{2}\cdot \varvec{u} \times \varvec{M}^\prime \right. \nonumber \\{} & {} \quad +\left. \frac{\Phi }{2} \cot {\left(\frac{\Phi}{2}\right)} \left( \varvec{M}^\prime - \varvec{u} \cdot (\varvec{M}^\prime \cdot \varvec{u})\right) \right] \nonumber \\\!\!\!\!\!\!\!\!\!= & {} \;\;\;\;\;\;\;\;\;\;\;\;\;\;\;\;\;\;\;\;\;\varvec{\omega }\cdot \varvec{M}\quad . \end{aligned}$$

Lastly, we write Eq. (18) in matrix form such that $\varvec{M}=\varvec{B}\cdot \varvec{M}^\prime =-\varvec{B}\cdot \partial U/\partial \varvec{\Phi }$:

19 $$\begin{aligned} \varvec{M}=&\left( \varvec{M}^\prime \cdot \frac{\varvec{\Phi }}{\Phi }\right) \cdot \frac{\varvec{\Phi }}{\Phi }\left( 1-\frac{\Phi }{2} \cot {\left( \frac{\Phi }{2}\right) }\right) \nonumber \\&+\frac{\varvec{\Phi }}{2}\times \varvec{M}^\prime +\frac{\Phi }{2} \cot {\left( \frac{\Phi }{2}\right) }\cdot \varvec{M}^\prime \nonumber \\ =&\left[ \underbrace{\left( \begin{matrix} 1 &{} -\frac{\phi _3}{2} &{} \frac{\phi _2}{2}\\ \frac{\phi _3}{2} &{}1 &{} -\frac{\phi _1}{2} \\ -\frac{\phi _2}{2} &{} \frac{\phi _1}{2} &{}1 \end{matrix}\right) }_{\varvec{B}_1}- \frac{1}{\Phi ^2} \left( 1- \frac{\Phi }{2} \cot \left( \frac{\Phi }{2}\right) \right) \right. \nonumber \\&\cdot \left. \underbrace{\left( \begin{matrix} \phi _2^2+\phi _3^2 &{} -\phi _1\phi _2 &{} -\phi _1\phi _3\\ -\phi _2\phi _1 &{} \phi _1^2+\phi _3^2 &{} -\phi _2\phi _3 \\ -\phi _3\phi _1 &{} -\phi _3\phi _2 &{} \phi _1^2+\phi _2^2 \end{matrix}\right) }_{\varvec{B}_2}\right] \cdot \varvec{M}^\prime , \end{aligned}$$

with $$\varvec{B} = \varvec{B}_1 - \frac{1}{\Phi ^2}\cdot \left( 1- \frac{\Phi}{2}\cdot \cot \left( \frac{\Phi}{2}\right) \right) \cdot \varvec{B}_2$$.

Eq. (19) holds true for all $\Phi \ne 0$. The cotangent term, however, can cause numerical problems for $\Phi \approx 0$. It is therefore recommended to approximate $\cot \left( \frac{\Phi }{2}\right) =\frac{2}{\Phi }-\frac{\Phi }{6}-\frac{\Phi ^3}{45\cdot 8}$ for small rotational displacements. With the first two terms, we already get a negligible discrepancy of only $1.5\cdot 10^{-11}$ to the theoretical value of $\cot \left( \frac{\Phi }{2}\right)$ for $\Phi =0.1^\circ$. For all three terms, pure numerical errors of ${\mathcal {O}}(10^{-13})$ occur. Due to further multiplications in the equation for $\varvec{B}$ with $\Phi$, the first two terms are sufficient to reach analytical deviations of or below the order of numerical errors. The low-angle approximation of $\varvec{B}$ is, thus, $\varvec{B} = \varvec{B}_1 - \varvec{B}_2/12$.

If we transform our data set for recorded torque output vectors values into $\varvec{M}^\prime = \varvec{B}^{-1}\cdot \varvec{M}$, we can derive the surrogate models for pure (negative) first-order partial derivatives of *U* with respect to angle components analogue to the three force components. Analogously, when predicting torques with a surrogate for the potential *U*, we have to multiply them with matrix $\varvec{B}$ in order to achieve a torque vector given in Cartesian coordinates.

## Appendix D: Generation of artificial data for training and evaluation of subject- and level-specific surrogate IVD models

In our previous study (Meszaros-Beller et al. 2023), we took a first step towards individualising a fully articulated generic thoracolumbar spine model (Hammer et al. 2022). For this, we extracted landmark positions of vertebra endplates for all spinal levels from the first thoracic vertebra T1 to the last lumbar vertebra (L5) and the pelvis from computed tomography scans of the Visible Male (VM) geometry (Spitzer et al. 1996). While Meszaros-Beller et al. (2023) used this geometric information to determine vertebra positions and orientations as well as ligament and muscle attachment sites for a MB spine model, we here created a detailed osseoligamentous FE model on the basis of the same landmark data set to complement the existing MB model in terms of individualisation of IVD characteristics. We thereby followed the procedure and adopted the model parameters from Little and Adam (2015). The osseoligamentous FE model served as a database for the training of IVD surrogates, which then have been implemented in the MB spine model.

In Appendix D.1, we describe the recording of multidimensional force-displacement data of all IVDs from the first thoracic (T1$\vert$2) to the last lumbar (L4$\vert$5) interspinal level. This way, we investigate the coupling of the elastic IVD responses in all six dimensions. Based on this data set, we created kernel models for each spinal level, which are capable of mimicking the multiaxial coupling in the mechanical characteristics, see Sect. 2.3.

Alongside with the kernel models, in order to compare their accuracy with commonly used bushing elements or low order polynomial approximations, we created second-, third- and fourth-order polynomial models for the L4$\vert$5 disc force potential as described in Sect. 2.2 based on the same training data set. Note that the second-order polynomial is equivalent to a common bushing element, i.e., a linear, symmetric stiffness matrix, with an offset force at the zero-displacement position of the joint. This offset can be interpreted as an initial displacement between the two bushing reference frames with respect to the relaxed state of the IVD, which is typically reached when both bushing frames coincide. The third- and fourth-order polynomial models for the force potential correspond to coupled second- and third-order polynomials, respectively, for the force and torque vectors as the order reduces by one according to Eq. (1).

Finally, the level-specific kernel models were implemented in the corresponding MB model of the VM geometry model in order to predict the subject’s elastic IVD responses during forward and lateral flexion movements, see Appendix D.2.

### D.1 FE simulations of subject- and level-specific IVDs

Starting from the geometric information of the VM spine anatomy gained in Meszaros-Beller et al. (2023), particularly the vertebral body (VB) shape, location and orientation, the entire spinal column from L5 to T1 was meshed using a custom python-script VirtuSpine to create an osseoligamentous FE model of the subject-specific spine according to the workflow described in Little and Adam (2015). The articulation of space enclosed by adjacent VBs was filled by detailed IVD models whose outer geometry was extrapolated from its adjacent vertebral endplates. By considering the vertebra endplates to match with the IVD endplates, every IVD was parameterised by eight points in each vertebra endplate plane. The centre of every endplate was chosen as a distinguished point and called endplate centroid (EPC). Furthermore, the endplate orientation was defined by its angles in the sagittal and the coronal plane as well as the axial rotation angle in the endplate plane.

Materials and parameters were adopted from Little and Adam (2015) and applied to all spinal levels. Being comprised of a hydrostatic inner part and a hyper-elastic outer ground matrix, the detailed IVD model accounted for the different mechanics of the nucleus pulposus and the annulus fibrosus, respectively. Embedded linear elastic ‘rebar’ elements represented the collagen fibres within the disc. Thus, the IVD’s biomechanical behaviour varied with interspinal level and subject-specific anatomy due to their individual dimensions that were dependent upon their corresponding endplate positions, orientations and shapes. Note, with this approach only the geometry determined the IVD characteristics while general material parameters remained unchanged.

As we were focussing on the IVD dynamics, the sixteen thoracolumbar IVD parts were segregated from the osseoligamentous FE model for further investigation. Every IVD was rotated and shifted into the average coordinate system of its endplates according to Appendix B, which is equivalent to the corresponding intervertebral joint coordinate system in the MB model of the VM, see Meszaros-Beller et al. (2023) (Appendix C). This way, the displacement loads, applied to the IVD endplates later, were already specified in the form of generalised coordinates in the kinematic chain. Further, it ensures comparable load scenarios between all discs in the subsequent simulations. On the other hand, it implies that a rotation about, e.g., the IVD’s local left-right-axis will not necessarily contribute to the global spinal forward flexion in the same way as local and global reference frames do generally not match.

We performed FE simulations applying loads onto single IVDs using the ABAQUS 6.14 software. Based on the assumption of rigid vertebrae in the MB approach, we considered the endplates to be non-deformable and connected all nodes on every endplate rigidly to their respective EPCs. Hence, the shape of all endplates was kept constant independent of the load applied. During all load steps, we fixed the inferior endplate, indicated by defining the *HOLD NODE* at the lower EPC. Displacement loads were applied to the *LOAD NODE* located at the arithmetic mean of both IVD EPCs in the initial position, i.e., the origin of the enplates’ average coordinate system. The *LOAD NODE* was rigidly connected to the superior EPC via a beam connector (see Fig. 5). Displacements denoted translations and rotations of the *LOAD NODE* with respect to its initial position in the intervertebral joint coordinate system. Additionally, the induced response forces of the IVD tissue onto its superior vertebra are recorded as summed forces—acting at the entire superior endplate—at the *LOAD NODE*. Due to the rigid link of the *LOAD NODE* to all nodes on the upper endplate, the acting force is identical in every of these nodes while the response torque only differs with lever arm.

As IVDs have an intrinsic pressure, we numerically increased the nucleus pulposus pressure by 0.25 kPa in twenty increments in the first load step of all simulations as in Little and Adam (2015), and enforced the IVD to return into its initial position in a second step before applying the displacement loads. Henceforth, we refer to this IVD state and geometry, rotated into the IVD’s respective joint coordinate system, and pre-strained by an intrinsic nucleus pressure, as the initial state or zero-displacement state of an IVD, which serves as the starting state for all following load scenarios. Note that IVDs in our approach are not in their rest state in the initial state.

To capture the focussed multiaxial, nonlinear and coupled mechanical responses, we recorded the six displacements and the accompanying six IVD response forces onto the superior vertebra not only during movements along 1) single DOFs, but also for 2) two coupled DOFs and for 3) motions in all six dimensions simultaneously. All analyses were performed as static load steps with direct user control of the intervertebral displacement and constant incrementation through the step. Every load scenario consists of a simulation starting in the initial state and ending at the target displacement vector, with linearly interpolated increasing displacements in every increment, followed by a equidistant, stepwise linear reduction back to the initial state.

Displacement loads were applied in a symmetric bounding box centred in the initial state with maximum displacement values $m_i$ for every DOF *i* chosen such that the FE simulations cover most of the physiological range of motion of an intervertebral joint, and such that the simulations can still converge. Precisely, the simulations were confined to a maximum displacement of $\pm 3~\textrm{mm}$ for anterior-posterior and lateral translation, the axial compression and extension were varied up to ±20% of the disc height, and we set the upper and lower boundaries for each rotational motion (frontal flexion-extension, lateral flexion-extension and axial rotation) to $\pm 9^\circ$ according to reported physiological limits from (Newell et al. 2017; Pearcy and Tibrewal 1984; Wang et al. 2013). This covers the full physiological range for all DOF except for the flexion-extension axis. According to Pearcy and Tibrewal (1984), the flexion range of motion is on average 15^∘^ in all lumbar levels with highest measured values of about 20/ 21^∘^ degrees. Subtracting the smaller value for the maximum extension, the flexion angle can still reach on average 13^∘^ in the L4$\vert$5 segment. For our force-displacement data base, we only used $\pm 9^\circ$ around the initial position which is 4^∘^ less than the full flexion angle. The reduced range was due to convergence issues with the FE model. Note that for overly thin thoracic and wedged IVDs the loading scenario had to be adapted, meaning that the $m_i$ were reduced, whenever the FE simulations were not converging. In the analysis of the VM disc mechanics, this was especially the case for the T4$\vert$5 IVD.

In load scenario 1), only one component *i* of the intervertebral joint state vector $\varvec{x}$ was altered while the remaining five components were fixed to zero. The value of the 1d displacement was increased up to the edge of the boundary box, by using increments of 1% of the target value to gain very dense training data along these trajectories.

The twelve single-DOF simulations of load scenario 1) imitate pure translations along and rotations about single unit axes up to their respective maximum value $m_i$ or down to their minimum value defined by $(-m_i)$. Executed with 100 increments each, this data set comprises 1201 different data points including the initial reference position. This load scenario allows to compare predicted with experimentally measured IVD force-displacement relations.

For every combination of two DOFs in load scenario 2), 20 simulations with ten increments each were performed with end positions $\pm (m_i,\pm m_j)$, $\pm (m_i,\pm m_j/2)$, $\pm (m_i/2,\pm m_j)$, $\pm (m_i,\pm m_j/3)$ and $\pm (m_i/3,\pm m_j)$ (for $i<j$), resulting in, in total, 3000 additional data points.

Furthermore, we acquired data for six-DOF motion trajectories in load scenario 3) using the MATLAB function *rand* to generate 200 random end positions in the 6d bounding box defined by the maximum absolute values $m_i$. Again using 10 increments per simulation, we gained 2000 independent data points. Before processing the data further, duplicate entries in the data set, stemming from the backward simulations, were deleted right away. Thus, in sum, 6201 different data points were recorded for every of the 16 IVDs and used to train the surrogate models. Additionally, we repeated the load scenario 3) with another 200 random 6d end positions in order to get a separate set of test data to validate the surrogate models in Sect. 3.1.

Starting from and ending at the initial position in every single load case results in a data-rich region around the zero displacement position. Consequently, we can expect the trained surrogate to be most precise in this state, which is also the initial position for the settling process under gravitation in our NMS MB simulations. We would like to note that neither the number of increments nor the movement velocity in the FE simulation had an influence on the IVD responses as the underlying FE model is based on an ideally elastic material. This implies that all recorded forces and torques do only depend on the actual translational and rotational displacement.

### D.2 Subject-specific thoracolumbar spine simulation to generate kinematic test data

We adopted the subject-specific MB model of the thoracolumbar spine for the VM anatomy from Meszaros-Beller et al. (2023) including all muscle and ligament threads as well as their parameters. This model was derived for the use with the MB simulation tool *demoa* (Schmitt 2022) and is already individualised compared to its baseline generic spine model (Hammer et al. 2022) with respect to the skeletal geometry and characteristic muscle and ligament lengths scaled to subject-specific attachment sites. In this work, we took the individualisation one step further by implementing level-specific IVD models between L5 and T1 taking into account the subjects’ geometry.

The complex, nonlinear characteristics of the detailed IVD models was condensed in the surrogates to the biomechanical function of an IVD in the spinal column. Particularly, this comprised the ability to resist the gravitational load and to restrict the range of motion of the—otherwise free—intervertebral joint by exerting response forces onto the endplates of its adjacent vertebrae. It was, therefore, sufficient to implement IVDs as force-torque-functions depending on the displacements in the intervertebral joint. In the acquisition of training data for the surrogate models (see Appendix D.1), we already ensured that the IVD reference frame located in the initial state of the *LOAD NODE* is equal to the corresponding joint reference frame (see Appendix B for details about the definition of local reference frames). Thus, we directly replaced the formerly used bushing elements by the developed kernel surrogate models from Sect. 2.3. Rotational displacements in the joints, typically parametrised by Cardan angles in *demoa*, were converted into an angle-vector convention to be in accordance with the angle description used as inputs for the surrogate models in the training data set. Predicted elastic response forces and torques were applied to the superior endplate and—with opposite sign—to the inferior endplate at the joint coordinate fixed to the superior endplate (analogue to the *LOAD NODE* in Appendix D.1) and continuously recalculated during the forward-dynamic MB simulations.

The thoracolumbar MB spine model with individualised IVDs is, afterwards, actuated such that it performs forward and lateral bending movements in order to investigate the accuracy of the surrogate models in typical spinal motions. These purely muscle-driven simulations were governed by a hybrid controller (Bayer et al. 2017), where the muscle stimulation *u* is determined by an open-loop contribution in combination with a feed-back mechanism regulating the muscle’s fibre length $l_\text {CE}$ to reach a target fibre length $\lambda$. A full set of such target fibre lengths for all muscles in the system defined a target position.

$$\begin{aligned} u=u_\text {open}+w\cdot \kappa \cdot \frac{l_\text {CE}-\lambda }{l_\text {CE,opt}} \end{aligned}$$

As in Meszaros-Beller et al. (2023), we chose the gain factor $\kappa =2.0$ and $u_\text {open}=0.04$ for back muscles and $u_\text {open}=0.02$ for all abdominal muscles in combination with an event-based switch between different target poses. The feedback controller was normalised by the muscle’s optimal fibre length $l_\text {CE,opt}$, and weighted by the factor $w=0.25$ throughout this study. The calculation of target lengths $\lambda$ is described in Rockenfeller et al. (2021) in more detail.

We performed whole spine simulations (see Fig. 6) following the procedure described in Meszaros-Beller et al. (2023) for the steady-state simulation to gain the equilibrated upright state under gravitational load and for the forward bending motion—merely with kernel surrogates included instead of generic bushing elements. As before, the initial position determined the zero-displacement state of all intervertebral joints, whereas the actual bending simulation starts from the equilibrated state. We performed a forward flexion with $2^\circ$-increments of lumbar angle in the intermediate target positions until a lumbar angle of $20^\circ$ was reached with respect to the equilibrated state ($\phi _\text {lum,0}^\text {flex} = 20.2^\circ$). Hereby, the lumbar angle was defined as the angular difference between the local cranial axes of the lowest (L5) and first lumbar vertebral body (L1) when projected onto the sagittal or frontal plane to determine the flexion or lateral angle, respectively (Meszaros-Beller et al. 2023). The simulations were stopped after the peak bending angle was reached.

In addition to the forward bending motion, we simulated left and right lateral bending starting from the settled position, in which we detected a slight tilt to the right with a lateral lumbar bending angle of $\phi _\text {lum,0}^\text {lat}=3.4^\circ$. Again, each lateral bending simulation started from the equilibrated position, and we chose target positions in $2^\circ$-increments of the lateral lumbar angle up to a maximum change in lateral lumbar bending angle of $\pm 14^\circ$ with ‘+’ indicating motions to the right and ‘–’ indicating left lateral bending. Note that the lumbar lordosis angle $\phi _\text {lum}$ as a typical representative for the evaluation of spinal curvature was measured between the vertebrae L1 and L5 using the angular difference in local longitudinal axis orientation, when projected onto the sagittal plane in case of the forward flexion angle $\phi _\text {lum}^\text {flex}$ and projected onto the frontal plane to gain the lateral flexion angle $\phi _\text {lum}^\text {lat}$.

During all three bending simulations, we detected rotational and translational displacements as well as reaction torques and forces calculated by the kernel surrogate in the L4$\vert$5 IVD every 10 ms. Afterwards, the recorded displacements were applied to the detailed FE model in ABAQUS 6.14 with 10 increments to reach every position. The results of the detailed model were used as reference values to determine the precision of the surrogate models.

## Appendix E: Comparison of detailed IVD model predictions with experimental data

The personalised and level-specific IVDs are a key feature accomplishing the geometric individualisation process of the generic model started in Meszaros-Beller et al. (2023). Using a detailed FE model to record coupled 6d data facilitates us to capture multidimensional kinetic coupling in the surrogate models. However, the biomechanical quality of the surrogates and, therewith, the significance of the MB simulation results depends on the validity of the underlying detailed IVD model. We, thus, evaluated the force-displacement relations of our subject-specific detailed model of the L4$\vert$5 disc with experimental values from cadaver studies in Fig. 7. Note that even though the elastic response of the IVD was determined on the individualised geometry of the Visible Male (Spitzer et al. 1996), its lumbar region can be considered healthy with a disc height of $16.6\textrm{mm}$ (measured between the geometric centres of the adjacent endplates in the joint reference frame, see Appendix B).

The obtained elastic IVD responses compare reasonably well with the experimental data of IVD reaction forces and torques (Tencer et al. 1982; Berkson et al. 1979; Schultz et al. 1979; Lin et al. 1978; Zhang et al. 2020) and stiffness (Costi et al. 2008) (see Fig. 7). This especially applies in the range of joint angles occurring in the bending movement simulations analysed in Sect. 3.2). However, particularly the shear forces are higher as compared to quasi-static data from Tencer et al. (1982), Berkson et al. (1979), Schultz et al. (1979) and Lin et al. (1978). In line with observations by Costi et al. (2008) and Amin et al. (2016) about a velocity dependent increase in stiffness of IVD and functional spinal unit, the material parameters of the detailed FE model use larger stiffness values corresponding to physiological movement speeds.

Note that the predicted IVD responses of the best polynomial (fourth order) and kernel surrogates differ from the detailed model. This has, however, only a minor effect on the biomechanical validity of the surrogates as deviations from their reference model are small compared to the absolute values, see Figs. 2 and 3.

## Appendix F: Verification of elastic surrogate models

In order to verify the development algorithm and implementation of the newly developed surrogates, we evaluated the gain and loss of energy. For this, we calculated the work done when travelling along a closed path through the 6d space of the intervertebral joint displacements, starting and ending at the same position. The net work done on this path should be zero for ideally elastic models (see Sect. 2.1).

Here, we exemplary depict the procedure for a 2d motion (see Fig. 8) and, determine the work starting from the initially modelled position with zero displacements, moving posteriorly by $2$ mm, then perform a pure extension motion (3^∘^ about the *y*-axis) followed by an anterior motion to $x=0$ mm, and, lastly, a forward flexion back to the initial state. By that, four path sections ($k=1,2,3,4$) are defined between two successive target points or a target and the initial/ end point.

As test models, we use two different kernel approximations to map the elastic coupled, nonlinear 6d force-torque pair as a function of the joint displacements for the same L4$\vert$5 data set, which was presented in Appendix D.1. The first surrogate model (S1) was developed mapping single force or torque components to the data with an algorithm similar to Haasdonk et al. (2021) extended to 6 dimensions of inputs and outputs, whereas the second model (S2) mapped a common force potential from which all force and torque components can be derived following the approach from Sect. 2.3.

As both surrogate models do not depend on velocity or time explicitly, we can arbitrarily choose a time of $t=1~\textrm{s}$ needed for every path section *p* ($p=1,2,3,4$), summing up to 4 s in total, and a discretisation of *N* equidistant displacement increments on every path section, such that the translational displacement $\Delta \textbf{x}_n$ (for $n=1,...,N$) and the displacement $\mathbf \omega _n\cdot \Delta t$, with the angular velocity $\omega _n$, remain constant within every path section. Assuming constant time intervals $\Delta t$, the net work $-\Delta U$ can be written in terms of the power $-\dot{U}$ and determined numerically using a simple rectangle method for integration,

21 $$\begin{aligned}- & {} \oint \textrm{d}\dot{U}(\textbf{ x},\mathbf { \Phi }) = -\sum _{n=1}^{4\cdot N} \frac{\Delta U(\textbf{x}_n,\varvec{\Phi }_n)}{\Delta t}\nonumber \\= & {} \sum _{n=1}^{4\cdot N} \left( \textbf{F}_n\cdot \frac{\Delta \textbf{x}_n}{\Delta t}+\textbf{T}_n\cdot \mathbf \omega _n\right) \end{aligned}$$

22 $$\begin{aligned}= & {} \sum _{p=1}^{4} \left( \frac{\Delta \textbf{x}_p}{\Delta t}\cdot \sum _{n=1}^{N} \textbf{F}_n + \mathbf \omega _p \cdot \sum _{n=1}^{N}\textbf{T}_n\right) . \end{aligned}$$

Note that Eq. (21) is independent of the chosen path and could be extended to movements with curved trajectories. The calculated power, however, scales linearly with the movement velocity, which becomes particularly relevant if the net power is not zero, i.e., for non-conservative surrogate models.

Figure 8 shows the expected behaviour for the calculated net power of both models. For the new kernel modelling approach S2, it decreases with finer discretisation steps, i.e., increasing step number *N* or higher accuracy in the integration solver. This illustrates that the observed net work performed is induced solely by the error of the rectangle integration method and vanishes for infinitely large *N* (or until the order of typical numerical errors of $10^{-16}$ is reached). In contrast, a model fit of single force and torque components leads to a loss or gain in mechanical energy—independent of the accuracy of the integration solver.

## Appendix G: Surrogate accuracy of single force and torque components

Analysing the precision of the different surrogate types developed in this study, we found the fourth-order polynomial and kernel approximation to perform best on training as well as test data with respect to relative errors of predicted force and torque vectors (see Sect. 3). A closer look at single components of the force-torque vector reveals strikingly high differences in relative errors between models but even amongst force and torque components predicted by the same surrogate (see Table 3). While lowest relative errors generally occur for the three components with largest absolute values, compressive force, lateral torque and flexion-extension torque, we see tremendous relative deviations for forces and torques with comparably small values throughout the movement trajectory. This is in accordance with the observation from Sect. 3.1, where the largest relative errors occurred at zero-crossings of the single force and torque components.

However, the trend of most force- or torque-displacement relations followed the one of the reference data as can be seen in Figs. 4, 9 and 10. Only the compressive force prediction of the second-order polynomial for both lateral bending motions, and the kernel prediction for the anterior-posterior shear force in case of the lateral movement to the right show a rather constant force-length relation, whereas the reference values decreased with increasing lumbar bending angle. The fourth-order polynomial provides the lowest maximum relative error in our test cases, whilst for the three main components, the kernel approximations achieved the, by far, best results with similarly low error values for the compressive force predicted by the third-order polynomial. For the latter, the other error values were, however, higher.

To conclude, comparably large relative errors occur for single components of the force-torque pair with low absolute values show (see Appendix G) while the error of the force or torque vector is low. As we saw in the single-DOF trajectories, this is mainly governed by large relative errors for low absolute values. Such a behaviour was expected as the surrogates were optimised for low absolute errors, not taking into account relative deviations in the modelling process. Different optimisation criteria might reduce these relative errors in the consideration of single force and torque directions.

**Table 3:** Average relative errors (in $\%$) of predicted force and torque vector components of all four surrogate models (second-, third- and fourth-order polynomial as well as kernel approximation) were evaluated for typical spine movements: forward flexion (FF), left (LLB) and right lateral bending (RLB). The three force components are the anterior-posterior (AP) shear, the lateral (LA) shear and the compressive (CO) force; the three torque components are lateral, extension (EX) and axial (AX) bending torque. Note that we allow for negative relative error values when the surrogate underestimates elastic responses

		AP shear	LA shear	CO force	LA torque	EX torque	AX torque
Second p	FF	$-$7.5	7.7	$-$4.2	4.3	8.4	18.3
	LLB	75.9	$-$29.8	$-$6.1	5.0	7.9	827.8
	RLB	552.1	46.5	$-$19.5	3.0	2.6	$-$48.5
Third p	FF	$-$32.1	11.4	0.6	18.2	16.2	0.8
	LLB	33.8	$-$30.2	3.0	9.3	13.4	832.5
	RLB	399.0	35.2	0.0	12.5	8.5	$-$29.7
Fourth p	FF	$-$35.2	9.1	$-$5.8	7.2	6.6	38.0
	LLB	32.5	$-$30.0	$-$4.9	$-$0.1	4.6	472.8
	RLB	356.0	18.2	$-$1.4	4.6	3.7	$-$26.2
Kernel	FF	$-$49.2	$-$60.3	$-$0.3	$-$0.8	$-$2.1	56.5
	LLB	26.9	$-$64.2	2.1	$-$0.3	$-$1.8	601.3
	RLB	830.5	$-$23.3	0.6	$-$0.6	$-$1.7	$-$52.7

## Footnotes

1 The explicit equations for this transformation depend on the angular representation chosen for $\varvec{\Phi }$, and the reader is referred to Appendix C for a detailed derivation of the equations and the corresponding pre- and post-processing steps.

2 https://gitlab.mathematik.uni-stuttgart.de/pub/ians-anm/ecvkoga
